# Exploring the phytochemical profile, antioxidant and anti-inflammatory potential of *Bidens pilosa*: A Systematic Review

**DOI:** 10.3389/fphar.2025.1569527

**Published:** 2025-08-01

**Authors:** Ekom Monday Etukudo, Ibe Michael Usman, Augustine Oviosun, Vivian Onyinye Ojiakor, Isxaq Abdi Jama, Wusa Makena, Danladi Makeri, Elna Owembabazi, Patrick Maduabuchi Aja, Josiah Ifie, Ilemobayo Victor Fasogbon, Victor Bassey Archibong, Emeka Anyanwu

**Affiliations:** ^1^Human Anatomy Department, Faculty of Biomedical Sciences, Kampala International University, Kampala, Uganda; ^2^ Department of Microbiology, Faculty of Biomedical Sciences, Kampala International University, Kampala, Uganda; ^3^Biochemistry Department, Faculty of Biomedical Sciences, Kampala International University, Kampala, Uganda; ^4^Human Anatomy Department, School of Medicine and Pharmacy, University of Rwanda, Kigali, Rwanda

**Keywords:** *Bidens pilosa*, cytoprotection, antioxidant properties, anti-inflammatory properties, systematic review

## Abstract

Medicinal plants have garnered significant attention for their potential in treating various human diseases. Many of these plants exhibit antioxidant and anti-inflammatory properties, which are crucial for mitigating the impact of oxidative stress and inflammation implicated in numerous clinical conditions. This review investigated the antioxidant and anti-inflammatory properties of medicinal plants, particularly *Bidens pilosa*, and their potential applications in disease management. A structured systematic approach was employed for this analysis. Scopus, PubMed, and Web of Science were searched using the following search algorithm: (“*Bidens pilosa*”) AND (“antioxidant”) AND (“anti-inflammatory” OR “anti-inflammatory”) on the second of April 2025 without any filters. At the end of the title, abstract and full text screening, only 50 articles met the inclusion criteria and hence included in the study. Most (35/50, 70%) were published within the years 2014–2024. Of the 50 studies, 23 (46%) were done in Africa, 14 (28%) in Asia, and 11 (22%) in South America. Most studies were done in a laboratory dish (29/50, 58%), with a smaller number done in animals (12/50, 24%). Fourteen percent (14%) of the studies used both *in vivo* and *in vitro* methods, and 4% were carried out on people. Out of the 50 studies, leaves were looked at most often (23 or 46%), followed by the whole plant (19 or 38%). *Bidens pilosa lowered the tissue levels of tumor necrosis factor (TNF)* and interleukin (IL)-6, IL-1β, and IL-8. It also improved the tissue levels of antioxidants glutathione while reducing lipid peroxidation via malondialdehyde (MDA). In conclusion, all the studies examined in the present study reported that *Bidens pilosa* possess antioxidant and anti-inflammatory potential, hence holding great promise in the management of oxidative stress and inflammation-related conditions.

## Graphical Abstract

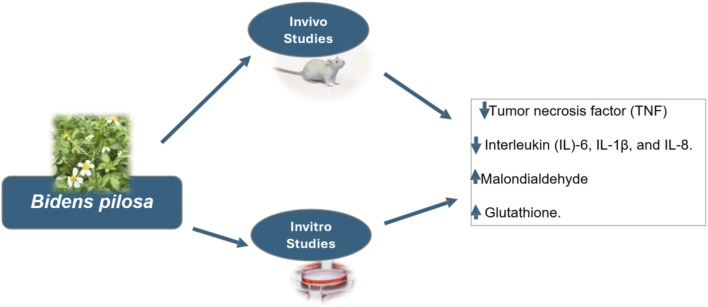

## 1 Introduction

Oxidative stress is implicated in the pathogenesis of various diseases, including Alzheimer’s disease, chronic obstructive pulmonary disease, cancer and atherosclerosis (Forman and Zhang, 2021). It is usually a consequence of an imbalance in the pro-oxidant and antioxidant ratio (Panfoli et al., 2018). Pro-oxidants are pro-biotics or xenobiotics that result in oxidative stress and its related damage; and could include free radicals or reactive oxygen species. Free radicals are associated with radical chain reaction, hence are referred to as oxidants (Tchekalarova and Tzoneva, 2023). Free radicals can be generated exogenously or endogenously; exogenous free radical generation can be through exposure to heavy metal, radiation exposure, alcohol consumption, and cigarette smoking (Ozsurekci and Aykac, 2016). On the other hand, the exogenous free radical generation can be triggered by stress, cancer, excessive exercise, immune cell activation, aging, ischemia, infection, and inflammation (Ozsurekci and Aykac, 2016).

Based on the fact that oxidative stress has been implicated negatively in various diseases, both natural and synthetic antioxidants have been increasingly recognized as promising therapeutic options (Daglia et al., 2014; Selamoglu-Talaz et al., 2013; Selamoglu et al., 2008; Sureda et al., 2023). Gallic acid and curcumin are examples of polyphenols that lessen the risk of multi-faceted mechanistic clinical conditions by reducing neuroinflammation and oxidative stress, consequently protecting against neurotoxicity (Daglia et al., 2014; Sureda et al., 2023). Phytomedicine has highlighted the antioxidant properties of plants such as *propolis* and synthetic organoselenium compounds (Selamoglu-Talaz et al., 2013; Selamoglu et al., 2008). Furthermore, through antioxidant and cholinesterase-inhibiting qualities, *Octaviania asterosperma*, *Scrophularia amplexicaulis*, and *Neurada procumbens* promote nerve regeneration and cognition (Hamedi et al., 2020; Rasul et al., 2019; Sevindik et al., 2021).

Inflammation is a complex biological response crucial for tissue repair and defense against pathogens. It involves distinct phases: an initial pro-inflammatory response mediated by the innate immune system, followed by a resolution phase characterized by a macrophage phenotypic switch and a return to tissue homeostasis through inflammatory cell apoptosis (Kulkarni et al., 2016). However, when dysregulated, inflammation can contribute to the pathogenesis of various pathologies (Kulkarni et al., 2016; Rauf et al., 2022; Teleanu et al., 2022). Natural and synthetic anti-inflammatory agents have been effective in preventing neuroinflammation, which is heightened in these pathologic conditions (Hamsalakshmi et al., 2022; Kalra et al., 2022; Kaur and Singh, 2022; Yousaf et al., 2022).

Most of these effective antioxidant and anti-inflammatory agents are derived from easily accessible and cultivable plants. This makes medicinal plants to gain significant global attention as therapeutic alternatives (Ayuba et al., 2022; Onohuean et al., 2021; Onohuean et al., 2024; Salihu et al., 2022; Usman et al., 2016; Usman et al., 2022; Yusuf et al., 2023).

*Bidens pilosa*, a plant native to South America but widely distributed worldwide, including Africa, China, Japan and the Americas, has emerged as a promising candidate due to its rich phytochemical profile (Bartolome et al., 2013). *Bidens pilosa* is rich in phytocompounds such as saponins, alkaloids, polyacetylenes, flavonoids, and diterpenes (Abdel-ghany et al., 2016). Studies have reported that *Bidens pilosa* possess anti-hyperglycemic, antiulcerogenic, antihypertensive, hepatoprotective, antipyretic, anti-malarial, anti-leukemic, anti-bacterial, anticancer, antioxidant, immunosuppressive and anti-inflammatory (Silva et al., 2011). In some parts of Uganda, the plant is used for wound healing; however, there is limited information or studies on specific phytochemicals to enable the development of useful products for the treatment and management of neurodegenerative diseases. Hence, the present review examined the antioxidant and anti-inflammatory potential of *Biden Pilosa*, highlighting this plant’s promising therapeutic potential for variable yet related disease conditions heightened by oxidative stress and inflammation.

## 2 Methodology

### 2.1 Search strategy

The antioxidant and anti-inflammatory potential of *Bidens pilosa*, was examined using a structured and systematic approach. Scopus, PubMed, and Web of Science were searched using the following search algorithm: (“*Bidens pilosa*”) AND (“antioxidant”) AND (“anti-inflammatory” OR “anti-inflammatory”) on the second of March 2024 without any filters.

### 2.2 Study selection criteria

The database (Scopus, PubMed, and Web of Science) search yielded 65 bibliographies and extracted as comma-separated value (CSV) files, saved, and merged. Duplicates were sorted and removed, the remaining documents were retrieved for title and abstract screening, followed by full-text screening and data extraction. Only peer-reviewed articles on the antioxidant and anti-inflammatory properties of *Bidens pilosa* published in English were included in the present review. At the end of the screening exercise, only 12 articles met the selection criteria, hence used for data extraction (Figure).

### 2.3 Quality assessment

The risk of bias assessment was done using the Systematic Review Center for Laboratory Animal Experimentation (SYRCLE) Checklist for the *invivo* studies (Hooijmans et al., 2014); on the other hand, Science in Risk Assessment and Policy (SCIRAP tool) as modified and used by Almeida et al. (2021) was adapted to assess the reporting quality for *invitro* studies since we could not find another tool (Roth et al., 2021). The findings from the risk of bias assessment were presented using the ROBVIS tool.

### 2.4 Data extraction

The data was extracted and classified as the following information: Author, Country, Study Design, Animals Number, participants or animal gender and strain, *invivo/invitro* experiments, disease model or cells used, part of the plant, extract/molecule, phytochemical analysis and key compounds identified, intervention on antioxidant and anti-inflammmatory potentials of *B. Pilosa* and study outcomes (Tables 1–3) and (Supplementary Tables S1–S3).

**TABLE 1:** Study characteristics and summarized data from articles reporting antioxidant and anti-inflammatory potentials of *Bidens pilosa*.

S/N	Study design	Plant part used	Animal or cells/Evaluation system	Gender/Age (wks)	Disease model	Country	References
1	*In vivo*	Not mentioned	Swiss mice	Male; 8–10	Intestinal mucositis	Brazil	Bastos et al. (2016)
2	*In vivo*	Leaves	*Mus musculus* mice and Wistar rats	Both sexes (mice 11–12 and rats 7–8)	Analgesic models (acetic acid-induced writhing, hot-plate, capsaicin-induced neurogenic pain, and formalin-induced), and anti-inflammatory models (carrageenan, dextran, histamine, and serotonin-induced paw edema)	Cameroon	Fotso et al. (2014)
3	*In vivo*	Whole plant	Wistar rats	Male Age not mentioned	Carrageenan-triggered paw swelling and arthritis induced by Complete Freund’s adjuvant	Taiwan	Chih et al. (1995)
4	*In vitro*	Whole plant	Mouse Mononuclear leukemia cells (RAW 264.7)	NA	Leukemia	China	Yan et al. (2022)
5	*In vitro*	Aerial part	Lipopolysaccharide (LPS)-stimulated RAW 264.7 macrophages and human leukemia monocytic cells (THP-1)	NA	Leukemia	Korea	Xin et al. (2021)
6	*In vivo* and *In vitro*	Leaves	Peripheral blood mononuclear cells (PMBC) and Murine Lymphocytes (*in vitro*); B10.ArSg SnJ mice (*in vivo*)	Not mentioned	Human lymphocyte proliferation; Murine lymphocyte proliferation; and Zymosan-induced arthritis in Mice	Brazil	Pereira et al. (1999)
7	*In vivo* and *In* *vitro*	Whole plant	Transgenic G93A Mice (with SOD 1 mutation)-*in vivo* and LPS-BV2 microglial cells	Not mentioned	Amyotrophic Lateral Sclerosis Mouse and Lipopolysaccharide (LPS)-Stimulated BV-2 Microglia	Japan	Tsuruta et al. (2023)
8	*In vivo*	Whole plant	Wistar/ST rats, ddY mice and BALB/c mice	Male, age not specified	Allergic response (IgE production), passive cutaneous anaphylaxis (PCA), inflammatory skin reactions, and histamine release from mast cells	Japan	Horiuchi and Seyama (2008)
9	*In vivo*	Aerial part	Wistar rats and Swiss mice	Male, age not specified	2,4,6-Trinitrobenzenesulfonic acid (TNBS)-induced colitis	Brazil	Quaglio et al. (2020)
10	*In vitro*	Whole plant	Oral squamous carcinoma cell line (SCC-4)	NA	Oral carcinoma	Brazil	Arantes et al. (2021a)
11	*In vivo*	Whole plant	BALB/c mice	Female; 6–8	Ovalbumin-induced Th2-mediated airway inflammation	Taiwan	Chang et al. (2005)
12	*In vivo* and *In vitro*	Whole plant	Rat cardiac microvascular endothelial cells (CMECs) from hearts of Sprague Dawley rats	Gender not specified for *In vitro* (1–3 days)	Rat model of Chronic Heart Failure; and Cultured Microvascular Endothelial Cells (CMEC) inflammation models	China	Y. Yang et al. (2018)
13	*In vivo*	Whole plant excluding the roots	Wistar rats	Adult males	Carbon tetrachloride (CCl4)-induced cardiac and hepato-toxicity	Brazil	Pegoraro et al. (2018)
14	*In vivo*	Not mentioned	Swiss mice	Male; 7–8	5-Fluorouracil (5-FU)-induced intestinal mucositis in mice	Brazil	de Ávila et al. (2015)
15	*In vivo*	Whole plant to formulate FITOPROT	Human	Male and Female, 18–65 years of age	Oral mucositis but the study included healthy participants with adequate bone marrow, liver, renal, glycemic parameters, and heart function, alongside health assessments such as oral intake and gingival indices, with participants abstaining from specific vegetal extracts during the study	Brazil	Santos Filho et al. (2018)
16	*In vivo and* *In vitro*	Whole plant	Wistar albino rats (*in vivo*), DPPH and ABTS radical scavenging assay (*in vitro*)	Male (*in vivo*)	Oxalate induced liver and kidney inflammation and toxicity	Egypt	Mohamed et al. (2024)
17	*In vitro*	Leaves	DPPH scavenging assay	NA	NA	South Africa	Akula and Odhav (2008)
18	*In vitro*	Whole plant	FRAP and DPPH assays	NA	NA	South Africa	Tesfay et al. (2016)
19	*In vitro*	Leaves	DPPH scavenging assay	NA	NA	Indonesia	Yuniastri et al. (2022)
20	*In vivo*	Whole plant	Wistar rats	Female; Age not specified	2,4,6 trinitrobenzene sulfonic acid (TNBS) induced colitis	Nigeria	Abiodun et al. (2020)
21	*In vitro*	Leaves	DPPH, FRAB and ABTS assays	NA	NA	South Africa	Nxumalo et al. (2023)
22	*In vitro*	Leaves	Bacteria: *S. typhimurium, S. boydii, V. parahaemolyticus,* and *E. coli/*DPPH assay	NA	Common diarrhea	South Africa	Shandukani et al. (2018)
23	*In vitro*	Leaves	DPPH, reducing power, and β-carotene-linoleate assays	NA	NA	Cameroon	Goudoum et al. (2016)
24	*In vivo and* *In vitro*	Whole plant	*In vivo*: BALB/c and C57BL/6 mice. *In vitro*: Hela cells or peritoneal cells from C57BL/6 mice. PMI-1640 medium supplemented with 25 mM HEPES, 2 mM L-glutamine, 100 U/mL penicillin, 100 μg/mL streptomycin	Male; 6–10	*Toxoplasma gondii* infected mice	Brazil	Mota et al. (2019)
25	*In vitro*	Leaves	DPPH, FRAP and hydrogen peroxide scavenging assays	NA	NA	Zambia	Phiri et al. (2024)
26	*In vitro*	Flower and leaves	ABTS and DPPH assays	NA	NA	Vietnam	Nguyen et al. (2023)
27	*In vitro*	Leaves	HepG2 cell line, derived from human hepatocellular carcinoma	NA	NA	Egypt	Said et al. (2024)
28	*In vitro*	Leaves, root, stem and whole plant	DPPH, ABTS and FRAP	NA	NA	Italy	Angelini et al. (2021)
29	*In vitro*	Leaves	DPPH, ABTS and Nitric oxide radical scavenging systems. Low Density Lipoprotein Oxidation system	NA	NA	Thailand	U-Yatung et al. (2020)
30	*In vitro*	Leaves	DPPH and FRAP and total antioxidant activity evaluating systems	NA	NA	Nigeria	Oduntan et al. (2018)
31	*In vitro*	Leaves	DPPH, ABTS and TBARS systems	NA	NA	South Africa	Falowo et al. (2017)
32	*In vitro*	Leaves	Human epidermoid carcinoma (KB-3-1) cells; DPPH and ABTS systems	NA	Cancer	India	Singh et al. (2017)
33	*In vitro*	Aerial part	HepG2 (cancerous), and Vero (non-can-cerous) cell lines; ABTS and FRAP systems	NA	Hepatocellular carcinoma	South Africa	Idris et al. (2023)
34	*In vivo*	Whole plant	Chickens	1 day	*Eimeria* infection	China	Memon et al. (2020)
35	*In vivo and* *In vitro*	Whole plant	Fruit fly (*Drosphilia melanogaster*); DPPH, ABTS, FRAP and Phosphomolybdenum total antioxidant activity systems	Males, 1–2 days	Paraquat induced oxidative stress	Vietnam	Son et al. (2022)
36	*In vitro*	Whole plant	*mycobacterium tuberculosis*; DPPH and ABTS	NA	Tuberculosis	South Africa	Mashinini et al. (2023)
37	*Invivo*	Whole plant to formulate FITOPROT	Human	Both genders; dentulous and edentulous	Oral mucositis caused by radio-chemotherapy was observed in patients with head and neck cancer (HNC) who had been referred for adjuvant or neoadjuvant RT/RCT treatment	Brazil	Arantes et al. (2021b)
38	*In vitro*	Leaves and flowers	DPPH and β-carotene bleaching methods	NA	Bacterial and fungal models	Japan	Deba et al. (2007)
39	*In vitro*	Whole plant	RAW 264.7 cells, Murine macrophage cell line; DPPH, Superoxide scavenging and Nitric oxide inhibition systems	NA	Inflammatory disease	Taiwan	Chiang et al. (2004)
40	*In vitro*	Whole plant	DPPH and ABTS	NA	Colonic cancer	China	Yi et al. (2016)
41	*In vitro*	Not mentioned	Radical scavenging systems on DPPH, Hydroxyl group, superoxide and ferric reducing systems	NA	Oxidative stress and cancer	Taiwan	Liu et al. (2013)
42	*In vitro*	Whole plant	DPPH, Reducing power, β-carotene-linoleate system and phospholipid peroxidation assays	NA	Oxidative stress and Lipid peroxidation model	Zimbabwe	Muchuweti et al. (2007)
43	*In vivo/In vitro*	Aerial part	Balb-c mice; *In vivo*: MDA using TBARS, Glutathione (GSH) and Catalase (CAT); *In vitro*: DPPH, Hydroxyl radicals, and FRAB	Male	Carbon tetrachloride (CCl4) induced lipid peroxidation and oxidative stress	Brazil	Kviecinski et al. (2011)
44	*In vivo*	Whole plant	ICR mice; TBARS system	Male	Lipid peroxidation and acute gastric mucosal lesion	Japan	Horiuchi et al. (2010)
45	*In vitro*	Leaves and shoots	DPPH radical scavenging, reducing power and β-carotene systems	NA	Oxidative stress	Zimbabwe	Chipurura et al. (2013)
46	*In vitro*	Whole plant	DPPH, BHT, FRAP and ABTS scavenging assays	NA	Oxidative stress	South Africa	Adeolu Adedapo and AuthorAnonymous (2011)
47	*In vivo*	Leaves	Mice and Sprague–Dawley rats; MDA, SOD and GSH-Peroxidase assays	Male	CCl_4_-induced liver damage and fibrosis	China	Yuan et al. (2008)
48	*In vitro*	Aerial part	DPPH, and ABTS radical scavenging assays	NA	Oxidative stress	Brazil	Cortés-Rojas et al. (2011)
49	*In vitro*	Whole plant	Antioxidant evaluation by DPPH, ABTS and FRAP assays; Cytotoxicity by MTT method	NA	Human tumor cells, namely MCF-7, HepG2, MGC 803 and RKO; Colorectal cancer	China	Wu et al. (2012)
50	*In vitro*	Whole plant	Human erythrocytes; MDA LPO evaluation using TBARS, Antioxidant evaluation using GSH and SOD	NA	Oxidative hemolysis and lipid/protein peroxidation of erythrocytes induced by the aqueous peroxyl radical [2,20-azobis(2-amidinopropane) dihydrochloride (AAPH)]	Taiwan	H. L. Yang et al. (2006)

NA- not applicable.

**TABLE 2:** Summarized data on intervention and dosages from included Studies.

S/N	Plant part	Phytocompound/extract or fraction	Dosage	Route of administration	Duration of administration	References
1	Not mentioned	Mucoadhesive or non-mucoadhesive formulations	75 mg/kg BP; 100 mg/kg BP; 125 mg/kg BP	Orally (gavage)	6 days	Bastos et al. (2016)
2	Leaves	Ethyl acetate fraction	50, 100 and 200 mg/kg	Topically	1–6 h	Fotso et al. (2014)
3	Whole plant	Aqueous extract	150, 300 and 500 mg/kg	Stomach tube	28 days	Chih et al. (1995)
4	Whole plant	Phytocompounds (1)4-O-b-D-glucopyranosyloxy-1-hydroxy- 6-(E)-tetradecene-8,10,12-triyne, (2) 3-O-b-D-glucopyrano-syloxy-1-hydroxy-6-(E)-tetradec- ene-8,10,12-triyne, (3) 2-O-b-D-glu-copyranosyloxy-1-hydroxy-5-(E)-tetradecene-7,9,11-triy-ene and (4) icthyothereol acetate	50 μg/mL	Cultured with cells	24 h	Yan et al. (2022)
5	Aerial part	Isookanin	1, 5, and 10 μg/mL	Cultured with cells	24 h	Xin et al. (2021)
6	Leaves	Methanolic extract and the polyacetylene [2-O-b-D-glucosyltrideca-11E-en-3,5,7,9-tetrayn-1,2-diol (PA-1)]	1, 5, or 10 μg/mL	Cultured with cells or intraperitoneal (i.p.) administration	cells- 72 h, Mice - 2–6 days	Pereira et al. (1999)
7	Not mentioned	Not mentioned	cells: 2–1000 μg/mL Mice: 2 g/kg/day	Cultured with cells or orally	cells- 72 h mice: 1 week	Tsuruta et al. (2023)
8	Aerial parts	Enzymatic digested *Bidens pilosa*	100, 250, and 500 mg/kg	Oral	10 days	Horiuchi and Seyama (2008)
9	Aerial parts	Nonpolar fatty acid enriched extract	25, 50, or 100 mg/kg	Orally	7 days	Quaglio et al. (2020)
10	Leaves	Not mentioned	0.01%–2%	Cultured with cells	24 h	Arantes et al. (2021a)
11	Not mentioned	Butanol fraction	10 mg/kg	Intra-peritoneal (i.p.) injections	35 days	Chang et al. (2005)
12	Whole plant	4-O-(2″-O-acetyl-6″-O- p-coumaroyl-β-D-glucopyranosyl) -p-coumaric acid	15, 30, and 60 mg/kg/d	Intragastric administration	8 weeks	Y. Yang et al. (2018)
13	Aerial parts	Bidens pilosa tea	orally; 0.5 mL of BP/100 g daily; topicaly, 500 mL	Oral and topical	10 weeks	Pegoraro et al. (2018)
14	Leaves	B. pilosa glycolic extract (Mucoadhesive formulation)	100 mg/kg	Orally (gavage)	6 days	de Ávila et al. (2015)
15	Not mentioned	B. pilosa glycolic extract (Mucoadhesive formulation)	15 mL	Gargle	10 days	Santos Filho et al. (2018)
16	Not mentioned	Aqueous extracts	100 mg/kg	Orally	21 days	Mohamed et al. (2024)
17	Leaves	Aqueous and methanolic extracts	NA	NA	NA	Akula and Odhav (2008)
18	Leaves	Perchloric acid extract	NA	NA	NA	Tesfay et al. (2016)
19	Leaves	Ethanol and aqua dest extracts	NA	NA	NA	Yuniastri et al. (2022)
20	Whole plant	Methanol extract	50,100, 200, and 400 mg/kg	Orally	9 days	Abiodun et al. (2020)
21	Leaves	50% methanol/1% formic acid	NA	NA	NA	Nxumalo et al. (2023)
22	Leaves	Hexane, dichloromethane, ethyl acetate, acetone and methanol	2.5–0.02 mg/mL	Culture	24 h	Shandukani et al. (2018)
23	Leaves	Distillated oil	NA	NA	NA	Goudoum et al. (2016)
24	Whole plant	Total extract and acetonic fraction	100 μg/mL or 300 μg/mL	Not mentioned	3 days	Mota et al. (2019)
25	Leaves	Crude methanolic and ethanolic extract	NA	NA	NA	Phiri et al. (2024)
26	Flowers and leaves	Water, methanol, acetone, and ethyl acetate	NA	NA	NA	Nguyen et al. (2023)
27	Leaves	DMSO extraction	500 μg/mL	Cell culture	Overnight	Said et al. (2024)
28	Leaves, roots, stems and whole plants	Methanol extract	NA	NA	NA	Angelini et al. (2021)
29	Leaves	Aqueous extracts	500 μg/mL	NA	NA	U-Yatung et al. (2020)
30	Leaves	Aqueous extracts	NA	NA	NA	Oduntan et al. (2018)
31	Leaves	Ethanol-water extract	NA	NA	NA	Falowo et al. (2017)
32	Leaves	Methanolic extract	1–10 mg/mL	Culture	36 h	Singh et al. (2017)
33	Aerial parts	Aqueous and ethanolic extracts	15.6, 31.25, 62.5, 125, 250, 500, and 1000 μg/mL	Cell culture	24 h	Idris et al. (2023)
34	Whole plants	NA	0.5 g/kg of feed	Orally	29 days	Memon et al. (2020)
35	Whole plants	Ethanol extracts	0.5 mg/mL	Medium	20 days	Son et al. (2022)
36	Whole plant	Water, acetone, methanol, hexane, and ethanol extracts	100 μg/mL	Culture	24 h	Mashinini et al. (2023)
37	Not mentioned	Mucoadhesive	10 mL	Gargle	Not mentioned	Arantes et al. (2021b)
38	Leaves and flowers	Essential oils from Bidens pilosa	NA	NA	NA	Deba et al. (2007)
39	Whole plant	Crude extract ethyl acetate and butanolic fractions	100, 200, 300, 400, 500 μg/mL	Cell culture	24 h	Chiang et al. (2004)
40	Whole herb	Petroleum ether, ethyl acetate (EE-BP), n-BuOH and water fractions	50, 100, 200 and 400 μmol/L	Cell culture	24 h	Yi et al. (2016)
41	NA	NA	NA	NA	NA	Liu et al. (2013)
42	Not mentioned	Aqueous methanol	NA	NA	NA	Muchuweti et al. (2007)
43	Aerial parts	Hydroethanol crude extract and chloroform, ethyl acetate, and methanol fraction	1.5, 15, 150, or 300 mg/kg	Not mentioned	10 days	Kviecinski et al. (2011)
44	Aerial parts	Suspension in 0.25% sodium carboxymethyl cellulose	0.1, 0.25, 0.5 mg/kg	Oral	3 weeks	Horiuchi et al. (2010)
45	Plant leaves and shoots	Methanolic extracts	NA	NA	NA	Chipurura et al. (2013)
46	Leaves	Acetone, methanol and aqueous extracts	0.1, 0.5, 1.0, 2.5 and 5.0 mg/mL	Cell culture	24–48 h	Adeolu Adedapo and AuthorAnonymous (2011)
47	Leaves	Total flavonoid extract	20, 50 and 100 mg/kg	Not mentioned	10 days	Yuan et al. (2008)
48	Aerial parts	Hydroalcoholic extract	NA	NA	NA	Cortés-Rojas et al. (2011)
49	Whole plant	Petroleum ether, ethyl acetate (EE-BP), and n-BuOH fractions	100, 200, 300, 400, 500 μg/mL	Cell culture	24 h	Wu et al. (2012)
50	Whole plant	Ethanol and ethyl acetate/ethanol extracts	50–150 and 25–75 ug/mL	Incubation	30 min	Yang et al. (2006)

NA- not applicable.

**TABLE 3:** Antiinflammatory potentials of *Bidens pilosa* as reported in the included studies.

S/N	Plant part, extract/phytocompound	Mechanism of action	Treatment outcome	References
1	NA	increased levels of IL-10 in intestinal tissue homogenate	Diminished inflammatory response	Bastos et al. (2016)
2	Leaf/methanol	Reduced the edema formation of the rat paw	Diminished inflammatory response	Fotso et al. (2014)
3	Whole plant	Decreased paw edema formation in rat paw	Inhibited paw edema	Chih et al. (1995)
4	Whole plant/ethanol	Reduced release of IL-6 and NO in RAW 264.7 cells	have anti-inflammatory activity	Yan et al. (2022)
5	flavonoids-type phytochemical, isookanin	Inhibited proinflammatory cytokines, interleukin-6, interleukin-8 and interleukin-1β. Inhibiting the expression of inducible nitric oxide synthase and cyclooxygenase-2	reduces the production of proinflammatory mediators (nitric oxide, prostaglandin E2) in mouse macrophages	Xin et al. (2021)
6	leaf/methanol	Inhibits the proliferative response of mouse lymphocytes	Possess anti-inflammatory and immunosuppressive activity	Pereira et al. (1999)
7	NA	Suppressed the induction of pro-inflammatory cytokine	Possess anti -inflammatory activity	Tsuruta et al. (2023)
8	Aerial parts/carboxy-methyl-cellulose sodium (CMC-Na) solution	Inhibits histamine release from mast cell pellet in peritoneal fluid supernatant	Possess antiinflammatory and antiallergic effects via the inhibition of histamine release from mast cells in peritoneal fluid supernatant of mice	Horiuchi and Seyama (2008)
9	Aerial parts/nonpolar fatty acid enriched extract	Inhibit IL-6, IL-1β and TNF-α and increment of anti-inflammatory IL-10 cytokine	Has anti-inflammatory activity and modulates the immune response	Quaglio et al. (2020)
10	mucoadhesive formulation	Reduced IL-8 production in an oral squamous cell carcinoma cell line (SCC-4), but had no observable effect on the production of the cytokines TNF-α, IL-6, IL-1β, IL-12p70, and IL-10 by the SCC-4 cell	exhibits anti-inflammatory properties by reducing IL-8 production in oral squamous cell carcinoma cell line (SCC-4)	Arantes et al. (2021a)
11	whole plants/butanol fraction	increased Th2 cytokines (IL-4 and/or IL-5), but decreased Th1 cytokine (IFN-c), increased Th2 cytokine-regulated IgE production in mouse serum	The butanol fraction of B. pilosa enhances Th2-mediated pulmonary inflammation by stimulating the production of Th2 cytokines and increasing IgE levels	Chang et al. (2005)
12	whole plants/ethanol	The inflammatory markers including tumor necrosis factor-α, (TNF-α), Interleukin-6 (IL-6), and Interleukin-1β (IL-1β) decreased	Suppress the expression of inflammatory mediators and effectively inhibit the inflammatory cytokines	Y. Yang et al. (2018)
13	Aerial parts/aqueous	10 high-power fields (HPF), Inflammatory cell present (polymorphonuclear and/or morphonuclear via presence in intestinal tissue. Presented mild inflammation	Absence of hepatic inflammation and inflammation in the intestinal mucosa	Pegoraro et al. (2018)
14	Glycolic extract	decreased MPO activities in 5-FU-induced intestinal mucositis in mice	Regulated inflammatory infiltration in the intestinal mucosa in mice via the inhibition of MPO enzyme activities	de Ávila et al. (2015)
15	Glycolic extract	Alteration in the concentrations of pro-inflammatory cytokines	No significant difference was found in the levels of pro-inflammatory cytokines between participants who used FITOPROT A versus FITOPROT B	Santos Filho et al. (2018)
16	Leaf/aqueous extracts	The kidney inflammation parameters TNF-α, IL 6, and TGF 1-β were analyze. All inflammation markers were notably declined in kidney homogenate of rats treated with BP extract compared to the untreated group	BP has an ability to reduce inflammation parameters in renal homogenates	Mohamed et al. (2024)
17	Leaf/Aqueous and methanolic extracts	B. Pilosa inhibit 5-lipoxygenase (5-Lox) activity	*Bidens pilosa* showed maximum antiinflammatory activity by inhibiting 5-lipoxygenase activity	Akula and Odhav (2008)
18	NA	NA	NA	Tesfay et al. (2016)
19	NA	NA	NA	Yuniastri et al. (2022)
20	whole plant/methanol	Myeloperoxidase activity (MPO), TNF-α were estimated in colon homogenate. BP decrease TNF-α and inhibited leukocytes infiltration (MPO activity)	BP significantly reduced leukocytes infiltration, and TNF-α level in comparison to untreated colitic rats	Abiodun et al. (2020)
21	Leaf/methanol extract	NA	NA	Nxumalo et al. (2023)
22	Hexane, dichloromethane, ethyl acetate, acetone and methanol	NA	NA	Shandukani et al. (2018)
23	leaf/methanol	NA	NA	Goudoum et al. (2016)
24	Whole plant/aqueous, Acetonic fraction	*Bidens pilosa* inhibits *T. gondii* via the lectin maturase K, disrupting protein synthesis in the parasite’s apicoplast, while maintaining low cytotoxicity to host cells	*B. pilosa* induce significant decrease of the parasite burden in brain tissue of *T. gondii*-infected mice. This suggests its potential as a therapeutic alternative for toxoplasmosis	Mota et al. (2019)
25	Leaf/aqueous	NA	NA	Phiri et al. (2024)
26	Flowers and leaves/water, methanol, acetone, and ethyl acetate	NA	NA	Nguyen et al. (2023)
27	Leaf/DMSO	The ELISA assay indicated that treatment with *B. pilosa* led to a significant increase in the pro-inflammatory cytokines IL-1α and IL-1β in response to HepG2 cells	*B. pilosa* extract exhibited potential inhibitory effects on Raf-1 and MEK-1 gene expression without causing detectable cytotoxicity, along with a significant reduction in autophagic activity following treatment	Said et al. (2024)
28	Leaf, roots, stems and whole plants/methanol	NA	NA	Angelini et al. (2021)
29	Leaf/aqueous	NA	NA	U-Yatung et al. (2020)
30	Leaf/aqueous	NA	NA	Oduntan et al. (2018)
31	Leaf/ethanol-water solution	NA	NA	Falowo et al. (2017)
32	Leaf/methanol	NA	NA	Singh et al. (2017)
33	Aerial parts/aqueous	NA	NA	Idris et al. (2023)
34	Whole plants	Pro-inflammatory cytokines (IL-6 and IL-8) aecal samples of chicks were assessed. BP upregulated IL-6 and IL-8	*B. pilosa* and probiotic + *B. pilosa* diets enhanced the activities of pro-inflammatory cytokines by increasing IL-6 and IL-8	Memon et al. (2020)
35	whole part/ethanol	NA	NA	Son et al. (2022)
36	whole plant/aqueous, acetone, methanol, hexane, and ethanol	NA	NA	Mashinini et al. (2023)
37	Not mentioned	Salivary concentrations of the pro-inflammatory cytokines were evaluated	Exhibits effective anti-inflammatory properties	Arantes et al. (2021b)
38	Leaves and flowers oil/diethyl ether	NA	NA	Deba et al. (2007)
39	whole plant/ethanol	Inhibited nitric oxide production in RAW 264.7 murine macrophage cell line	Showed strong antiinflammatory activity in cells	Chiang et al. (2004)
40	whole plan/ethanol	NA	NA	Yi et al. (2016)
41	Honeys from the nectar of BP	Cytokine IL-8 of human colon carcinoma cell line (WiDr) was evaluated. *B. pilosa* exhibited low inhibition for IL-8 secretion by the WiDr	Immunomodulatory effects of the honey from BP seem to be attributed to components other than phenols as honey from *B.pilosa* had the lowest inhibition of IL-8 production by WiDr cells	Liu et al. (2013)
42	Methanol	NA	NA	Muchuweti et al. (2007)
43	Aerial parts/ethanol-water	NA	NA	Kviecinski et al. (2011)
44	Aerial parts/sodium carboxymethyl cellu-lose (CMC-Na)	MMBP reduced severity of gastric lesions induced by NSAID	Possess suppressing effects against the NSAID-induced gastric lesion model via reducing severity of gastric lesions	Horiuchi et al. (2010)
45	Leaf/total phenolic content	NA	NA	Chipurura et al. (2013)
46	Leaf/acetone and methanol	NA	NA	Adeolu Adedapo and AuthorAnonymous (2011)
47	Leaf/ethanol	nuclear factor-B (NF-B) expression of the liver were assessed. Marked reduction in the NF-B protein expression in rat hepatocytes	Exhibited NF-B activation inhibition property by B. Pilosa inhibited NF-B activation in liver fibrosis of rats	Yuan et al. (2008)
48	Aerial parts	NA	NA	Cortés-Rojas et al. (2011)
49	whole plant/ethanol	NA	NA	Wu et al. (2012)
50	whole plant/ethanol	NA	NA	H. L. Yang et al. (2006)

NF-κB: Nuclear Factor kappa-light-chain-enhancer of activated B cells; IL-8: Interleukin 8; IL-6: Interleukin 6; IL-1β: Interleukin 1 beta; IL-1α: Interleukin 1 alpha; TNF-α: tumor necrosis factor alpha; COX-2: Cyclooxygenase-2.

## 3 Result

### 3.1 Study selection

**FIGURE 1:**
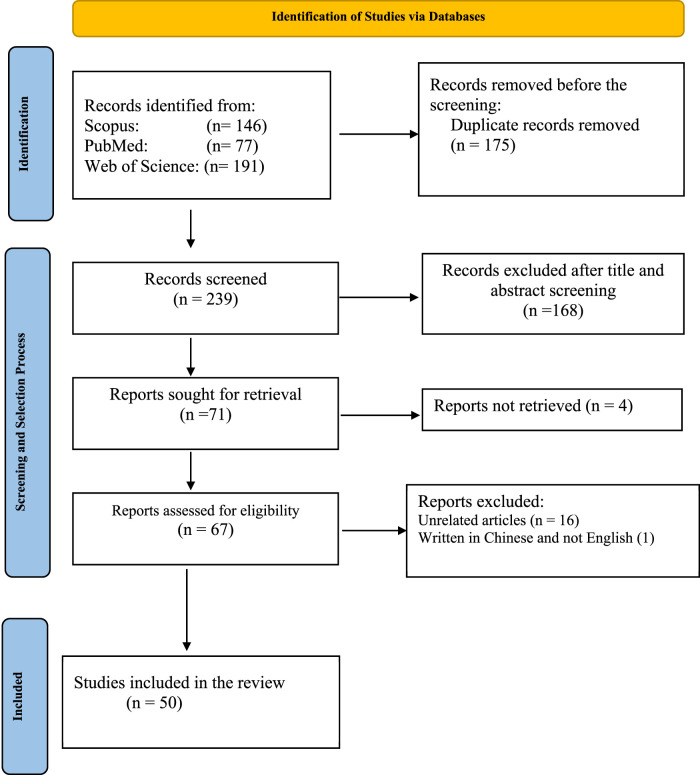
Prisma flow chart.

Our search of three database searches returned 414 bibliographies from which 175 duplicates were removed. The remaining 239 articles were used for title and abstract screening, at the end of which 168 articles were excluded. The report sorted for retrieval was 71, out of which we were unable to retrieved 4 articles despite all our efforts. The reports assessed for eligibility were 67, out of which 16 articles were excluded for been unrelated and 1 article for been written in Chinese. At the end of the entire screening process, 50 articles were included in the study for data extraction and synthesis (Figure 1).

### 3.2 Risk of bias assessment

**FIGURE 2:**
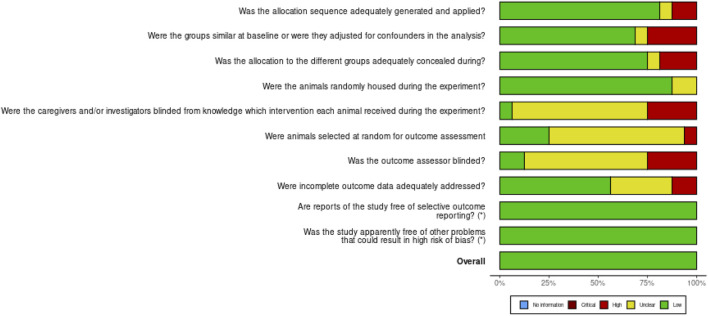
Risk of bias for the 10 domains for the *in vivo* studies.

**FIGURE 3:**
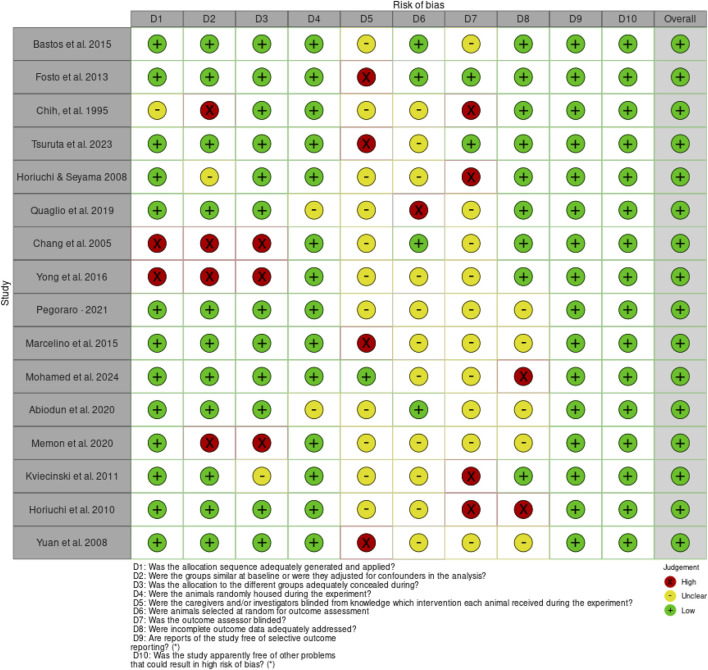
Risk of bias for the individual articles on the *in vivo* studies.

The risk of bias assessment using SYRCLE Checklist for the *in vivo* studies based on the 10 domains revealed a general low risk of bias (Figure 2); however, 11 articles had no information about “D5 - Were the caregivers and/or investigators blinded from knowledge which intervention each animal received during the experiment?” and “D6 - Were animals selected at random for outcome assessment?”. Ten articles had no information on “D7 - Was the outcome assessor blinded?” (Figure 3).

**FIGURE 4:**
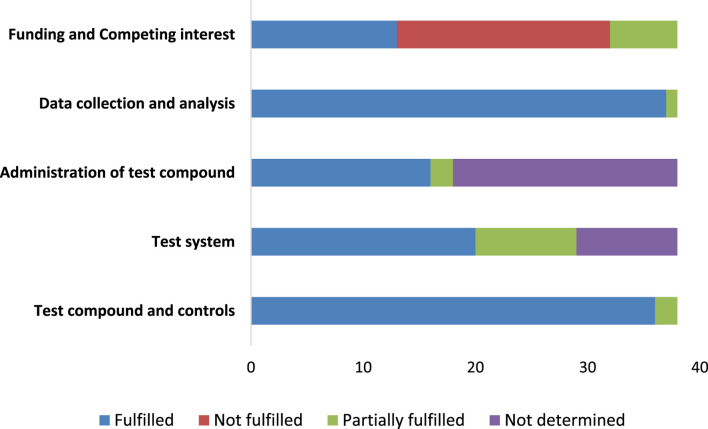
The reporting quality for the *in vitro* studies.

The SciRAP tool evaluation covered five key components: test compound and controls, test system, administration of the test compound, data collection, and analysis, encompassing a total of 23 topics. In assessing the test compound and controls, 36 studies were found to be partially fulfilled. For the test system and administration of the test compound, 20 studies met the criteria. Data collection and analysis were fulfilled in 37 studies. Regarding funding and competing interests, 13 articles met the criteria, while 19 did not (Figure 4).

### 3.3 Study characteristics

We reviewed 50 studies, and most (35/50, 70%) were published within the years 2014–2024. Of the 50 studies, 23 (46%) were done in Africa, 14 (28%) in Asia, and 11 (22%) in South America. Most studies were done in a laboratory dish (29/50, 58%), with a smaller number done in animals (12/50, 24%). Fourteen percent (14%) of the studies used both *in vivo* and *in vitro* methods, and 4% were carried out on people. Out of the 50 studies, leaves were looked at most often (23 or 46%), followed by the whole plant (19 or 38%). Most of the studies (44/50, 88%) concentrated on the roots, stems, or leaves, but the aerial parts were studied in just 4/50 (8%) of the studies (Table 1). To study antioxidant, anti-inflammatory, analgesic, anticancer and immunomodulatory effects, the researchers used both living and non-living models. For mucositis and oxidative stress, male Swiss mice (7–10 weeks) were used *in vivo*; for TNBS-induced colitis, carrageenan-induced edema and CCl_4_-induced toxicity, Wistar rats and BALB/c mice were used. Ovalbumin-induced airway inflammation was studied in female BALB/c mice, while transgenic G93A mice represented ALS. Chickens were used in Eimeria infection models and *Drosophila* were used to study the effects of paraquat on oxidative stress. RAW 264.7, THP-1, BV-2, SCC-4, HepG2, Vero, HeLa and KB-3-1 cell lines were used in in vitro systems to investigate leukemia, inflammation and several cancers. Both healthy volunteers and patients who had oral mucositis while receiving radio-chemotherapy were studied in this research. The antioxidant property and toxicity of the extracts were measured by DPPH, ABTS, FRAP, TBARS, SOD, GSH, catalase, β-carotene bleaching, phosphomolybdenum and MTT assays. They gave a complete understanding of oxidative stress, the immune system, inflammation and cancer development.

### 3.4 Summary of intervention and dosages of *Bidens pilosa* from the included studies

The included studies used different preparations of *B. pilosa* such as aqueous, methanolic, ethanolic and glycolic extracts, as well as isolated phytocompounds and essential oils. Most of the plants used were leaves, aerial parts and whole plants (Table 2). The amount and frequency of the extracts were different for each type of extract and model in the studies. In animals, researchers most often gave the compound via gavage or feed and doses varied from 15 mg/kg to 2 g/kg each day for up to 10 weeks (Bastos et al., 2016; Abiodun et al., 2020; Yang et al., 2018). In a few studies, intraperitoneal injection was used, with a 10 mg/kg butanol fraction given for 35 days (Chang et al., 2005). The plant was given to patients in human clinical trials either as a gargle (15 mL for 10 days) or in the form of a mucoadhesive (at 100 mg/kg by mouth) (Santos Filho et al., 2018; de Ávila et al., 2015) (Table 2). *In vitro* studies most often used *Bidens pilosa* phytocompounds at concentrations between 1 μg/mL and 1000 μg/mL and left the cells in contact with them for 24–72 h (Xin et al., 2021; Pereira et al., 1999; Chang et al., 2005). Interestingly, some research used mixtures of phytochemicals, for example, glucopyranosylated polyacetylenes (Yan et al., 2022) and essential oils (Deba et al., 2007), but did not always mention how much or for how long the compounds were given. Plant extracts were also used in foods or as ingredients in teas, much like they have been used in the past (Pegoraro et al., 2018). Less specific detail on how the botanical drugs were given was observed in some studies (Akula and Odhav, 2008; Yuniastri et al., 2022).

### 3.5 Phytochemical analysis of *Bidens pilosa* extracts and key phytocompounds identified

A variety of methods have been used to study the phytochemical content of *Bidens Pilosa* (Supplementary Table S1). Ultraviolet-Visible spectrophotometry (UV-Vis Spec.) was widely used to measure the total phenolic and flavonoid content, as shown by Mohamed et al. (2024), Akula and Odhav (2008) and Phiri et al. (2024). Specific phenolic acids and flavonoids were identified and quantified using HPLC, as was done by Nguyen et al. (2023), Yang et al. (2018) and Singh et al. (2017) (Supplementary Table S1). Using LC-MS/MS and LC-HR-MS, researchers were able to identify a wider range of secondary metabolites such as coumarins, ellagitannins and hydroxybenzoic acids (Angelini et al., 2021; Idris et al., 2023; Nxumalo et al., 2023). GC-MS was found to be the best method for detecting fatty acids and terpenoids among volatile and lipophilic compounds (Goudoum et al., 2016; Abiodun et al., 2020; Falowo et al., 2017) (Supplementary Table S1).

The included studies found that *B. pilosa* contains many bioactive compounds, suggesting it may be useful in medicine. The phytocompounds are shown in Supplementary Table S2. Quercetin, isookanin, rutin, and glucuronylated quercetin were found in every extraction method studied by Fotso et al. (2014), Xin et al. (2021), and Santos Filho et al. (2018). Ethyl acetate and n-butanol extracts from the plant were commonly found to contain 4-O-β-D-glucopyranosyloxy-1-hydroxy-6-(E)-tetradecene-8,10,12-triyne (Yan et al., 2022; Chang et al., 2005) (Supplementary Table S2). Caffeic acid, gallic acid, and chlorogenic acid were other phenolic acids that were commonly found, according to Horiuchi and Seyama (2008) and Nguyen et al. (2023). GC-MS tests also found that the main fatty acids and volatile oils in these oils are palmitic acid, oleic acid, α-pinene, and phytol (Quaglio et al., 2020; Goudoum et al., 2016) (Supplementary Table S2). They highlight the plant’s rich chemical makeup and its ability to help with diseases linked to oxidative stress, lipid peroxidation, and inflammation.

### 3.6 *Bidens pilosa* antioxidant mechanisms and outcome as reported in the included studies

It was shown that *Bidens pilosa* acts as a powerful antioxidant by removing free radicals and helping the body produce its own antioxidants (Supplementary Table S3). Methanolic, aqueous, ethanolic and ethyl acetate extracts from the plant showed strong DPPH, ABTS and FRAP scavenging abilities, similar to those of ascorbic acid and BHT (Chipurura et al., 2013; Adeolu Adedapo and AuthorAnonymous, 2011; Cortés-Rojas et al., 2011; Wu et al., 2012). There was a strong relationship between these activities and the total phenolic and flavonoid contents. In addition to neutralizing radicals, *B. pilosa* changed the activity of SOD, GSH and GSH-Px which resulted in a decrease in lipid peroxidation and MDA levels in both hepatic and erythrocyte models (Yuan et al., 2008; Yang et al., 2006). The gastric models showed that its formulations greatly reduced thiobarbituric acid reactive substances (TBARS) which indicated that it helped protect the stomach by reducing oxidative stress (Horiuchi et al., 2010) (Supplementary Table S3). In therapeutic terms, these antioxidant properties resulted in protecting cells from damage, including the liver, stomach and colon cancer cells (Wu et al., 2012). In general, the powerful ability to scavenge radicals, alter enzymes and provide protection make B. pilosa a promising natural antioxidant for disease prevention and management. All the studies included in this review have shown that Bidens Pilosa has beneficial antioxidant properties (Supplementary Table S3). A formulation of *B. pilosa* (MMPB) minimized oxidative markers such as MDA and TBARS, improved antioxidant enzymes (SOD, GSH, GSH-Px) and reduced tissue damage in models of liver, stomach and erythrocyte injury (Horiuchi et al., 2010; Yuan et al., 2008; Yang et al., 2006). Radical scavenging tests revealed that methanolic, ethanolic and ethyl acetate extracts have potential for treating conditions related to oxidative stress (Chipurura et al., 2013; Adeolu Adedapo and AuthorAnonymous, 2011; Wu et al., 2012). The ethyl acetate fraction caused cancer cells to die which suggests it may be useful in treating colorectal cancer. These reports reveal that *B. pilosa* can be used in various ways as an antioxidant and has many therapeutic benefits.

### 3.7 *Bidens pilosa* anti-inflammatory mechanisms and outcome as reported in included studies

Anti-inflammatory activity in *Bidens pilosa* has been shown in many studies using different plant materials and types of extracts (Table 3). Bastos et al. (2016), Quaglio et al. (2020), Pegoraro et al. (2018), and de Ávila et al. (2015) found that *B. pilosa* affects cytokines, boosting IL-10 and lessening IL-1β, IL-6, and TNF-α in tissues affected by inflammation. The anti-inflammatory and immunosuppressive effects of leaves and whole plants from this plant were confirmed by reducing paw edema, lymphocyte proliferation, and histamine release in animal experiments (Fotso et al., 2014; Chih et al., 1995; Pereira et al., 1999; Horiuchi and Seyama, 2008). Experiments done in a laboratory have found that it is effective. Yan et al. (2022) and Yang et al. (2018) found that ethanolic extracts of *B. pilosa* reduced IL-6, NO, and TNF-α in macrophages, while isookanin flavonoids from the plant blocked COX-2 and iNOS enzymes (Xin et al., 2021) (Table 3). According to Tsuruta et al. (2023), they discovered that pro-inflammatory cytokines in the spinal cord were selectively blocked in G93A mice. Meanwhile, Santos Filho et al. (2018) reported no changes in human inflammation, which suggests that clinical application varies. The reports show that *B. Pilosa,* helps reduce inflammation by controlling cytokines, limiting inflammatory mediators, and regulating the immune system.

Experiments across multiple models showed that *B. pilosa* has anti-inflammatory effects. The outcome revealed that extracts help reduce edema in acute inflammation, decrease inflammatory cytokine levels in both the intestine and kidney, and decrease the number of leukocytes and MPO activity in colitis models (Fotso et al., 2014; Chih et al., 1995; Yang et al., 2018; Mohammed et al., 2024; Abiodun et al., 2020) (Table 3). The treatment decreased IL-8 release in oral carcinoma (Arantes et al., 2021a). Specifically, the plant’s ability to fight colitis was linked to higher IL-10, lower IL-6, and lower TNF-α levels (Quaglio et al., 2020). Mucoadhesive gels showed advantages for certain tissues while still keeping the overall cytokine profile unchanged (as shown by Santos Filho et al., 2018) (Table 3).

## 4 Discussion

### 4.1 Study characteristics and scientific methodological overview

This review looks at fifty studies on *Bidens Pilosa (B. pilosa)*, mainly focusing on its antioxidant, anti-inflammatory, and immunomodulatory effects. Most of the studies, 70%, were published during 2014–2024, which points to a growing interest worldwide in using this plant as a botanical drug because of its important metabolites and many medical uses. Significantly, studies from Africa, Asia, and South America confirm that people from different regions recognize how bioactive this plant and its metabolites can be.

This data shows studies ranging from *in vitro* designs to *in vivo* experiments, while some studies combined the two approaches. Although currently in an early translational phase, the few human clinical trials (Arantes et al., 2021a; Santos Filho et al., 2018) form a solid base for future studies. These results agree with previous studies (Abiodun et al., 2020; Rodríguez Mesa et al., 2023), highlighting the importance of bringing preclinical results into clinical use.

Although preclinical studies prove that *B. pilosa* has antioxidant and anti-inflammatory properties, very few clinical trials have been done. The lack of translation may be caused by problems in the way this plant is extracted, the variation in doses, and the different active compound concentrations found in studies, making it hard to standardize how cannabis should be used in humans. In addition, the information from preclinical studies is usually not robust enough in pharmacokinetics and toxicity to get ethical approval for clinical trials. Limits on regulations, money, and the belief that herbal remedies are not scientifically proven hold back progress to human studies. Future work should focus on standardizing *B. pilosa* extracts to guarantee that results from different studies are the same. Immense pharmacological and toxicological evaluations can strengthen the drug design and safety, thereby helping it gain approval from regulators.

In this current review, tests using Swiss mice for intestinal mucositis, oxidative stress, and inflammatory bowel models in BALB/c and Wistar rats, as well as transgenic mouse models of ALS, show the wide range of conditions studied with *B. pilosa*. Furthermore, the choice of RAW 264.7 (macrophages), THP-1 (monocytes), BV-2 (microglia) and SCC-4 (oral cancer) cell lines demonstrates how the plant relates to immunological, oxidative, and oncogenic pathways.

The results also highlight that *B. pilosa* is a multi-purpose botanical drug. In particular, leaves and whole plant extracts were most studied, pointing to their high content of bioactive substances. This matches earlier research that found the leaves’ extracts in water and methanol could scavenge radicals (Akula and Odhav, 2008; Fotso et al., 2014) and whole plant extracts helped treat inflammation and oxidative damage in colitis and hepatic toxicity (Abiodun et al., 2020; Mashinini et al., 2023). Despite this, phytochemical analysis of the aerial parts (8%) and roots/flowers has yet to be done, and these plant parts should be studied further.

In all these experiments, DPPH, ABTS, TBARS, and FRAP were chosen to evaluate radical scavenging activity. MTT, catalase, and GSH assays were used to measure how the compounds affected cells and enzymes. Together, these assays measure the plant’s capacity to clear free radicals, control redox balance, and reduce the release of inflammatory cytokines, all of which are important for many diseases (Rodríguez Mesa et al., 2023; Santos Filho et al., 2018).

These findings have important consequences. Since *B. pilosa* can affect many biological systems and diseases, it is a good candidate for making multi-targeted phytotherapeutics. Because it works well in both immune and oxidative models, it is well suited to be a dual-action agent, which is important when diseases negatively impact multiple biological pathways. As a result, we require botanical drugs that treat the main condition and its linked problems, such as oxidative stress, which is found in many chronic inflammatory and neurodegenerative diseases.

### 4.2 Intervention strategies and dosage patterns in *Bidens pilosa* administration

The results from this review show a lot of variation in how *B. pilosa* was studied and given to participants. Such variety demonstrates the plant’s many uses and the absence of a set way to use it in preclinical and clinical research.

Different extracts of *B. pilosa* were used in the studies. For example, aqueous, methanolic, ethanolic, glycolic, and butanolic fractions, isolated phytocompounds, and essential oils. Most studies used leaves, aerial parts, or the whole plant to make interventions, reflecting the multiple therapeutic components in *B. pilosa* (Bastos et al., 2016; Abiodun et al., 2020; Chang et al., 2005; Deba et al., 2007). The results add to earlier studies showing that *B. pilosa* contains many metabolites, especially polyacetylenes, flavonoids, and phenolic compounds, which have been found to have various biological activities (Yan et al., 2022; Xin et al., 2021).

In animal experiments, the majority of researchers gave *B. pilosa* by gavage or mixed it into the feed, using amounts from 15 mg/kg to 2 g/kg each day for periods of 6 days–10 weeks (Yang et al., 2018; Abiodun et al., 2020). Since the best dose is not feasible, the broad range of doses used in research makes it challenging to see how effective the treatment is in various models. Significant outcomes were obtained by Chang et al. (2005) when they treated mice with a butanol fraction at 10 mg/kg daily for 35 days by injection into the abdomen. Since drugs are delivered and stay in the body differently, pharmacokinetic research is done to determine the best doses and improve outcomes.

The majority of *in vitro* studies used extracts at concentrations from 1 to 1000 μg/mL for 24–72 h (Pereira et al., 1999; Chang et al., 2005; Xin et al., 2021). Even so, the findings indicate that *B. pilosa* metabolites affect cells in a way that depends on both the dose and the time, even though a lack of standard endpoints prevents direct comparison between studies. Furthermore, research with complex mixtures, like glucopyranosylated polyacetylenes (Yan et al., 2022) and essential oils (Deba et al., 2007), often failed to clearly state the amounts and lengths of exposure, which weakens their reproducibility and usefulness for translation.

Fortunately, research on humans, while limited, has shown that clinical use is possible. Santos Filho et al. (2018) showed that a *B. pilosa* gargle (15 mL/day for 10 days) could be therapeutic, and de Ávila et al. (2015) gave 100 mg/kg orally in the form of a mucoadhesive preparation. They are especially important because they look at how these approaches are delivered and whether people will accept them in practice. Even so, the limited number of patients, short follow-up, and lack of pharmacokinetic data weaken these results and suggest that further studies are needed.

Our results are consistent with earlier reviews and analyses, which confirm that *B. pilosa* has broad bioactivity and may be developed as phytopharmaceutical agents (Pegoraro et al., 2018; Pereira et al., 1999). Still, the differences found and the challenges in methods suggest that we need strong standardization and better ways to move from research to clinical use.

### 4.3 *Bidens pilosa* phytochemical analysis, composition, and pharmacological importance

The phytochemical analysis of *Bidens pilosa* presented in this review confirms the plant’s status as a chemically diverse medicinal herb with high therapeutic potential. Through various analytical platforms and extraction methodologies, researchers have consistently identified a spectrum of secondary metabolites, including flavonoids, phenolic acids, polyacetylenes, fatty acids, and terpenoids. These findings provide a robust chemical foundation to support *B. pilosa*’s ethnomedicinal applications in managing oxidative stress, lipid peroxidation, inflammation, and metabolic disorders.

The repeated use of UV-visible spectrophotometry across studies, such as those by Mohamed et al. (2024), Akula and Odhav (2008), and Phiri et al. (2024), has facilitated rapid quantification of total phenolic and flavonoid content, which are important classes of antioxidants. These bulk metrics serve as initial indicators of pharmacological relevance, given the well-established role of flavonoids and phenolics in neutralizing free radicals and mitigating oxidative damage.

More targeted profiling was achieved by High-Performance Liquid Chromatography (HPLC), which allowed the specific identification and quantification of key compounds such as caffeic acid, chlorogenic acid, and quercetin derivatives (Nguyen et al., 2023; Yang et al., 2018; Singh et al., 2017). These phenolic acids and flavonoids are recognized for their antioxidant, anti-inflammatory, and vasoprotective activities, suggesting that *B. pilosa* could exert multifaceted biological effects relevant to chronic inflammatory diseases.

Advanced metabolomic platforms like Liquid Chromatography–Tandem Mass Spectrometry (LC-MS/MS) and Liquid Chromatography–High-Resolution Mass Spectrometry (LC-HR-MS) revealed an even broader spectrum of bioactive constituents, including coumarins, ellagitannins, and hydroxybenzoic acids (Angelini et al., 2021; Idris et al., 2023; Nxumalo et al., 2023). The identification of such structurally diverse molecules underscores the plant’s chemotypic complexity and supports its potential utility in drug discovery pipelines. Notably, polyacetylenic glucosides, especially 4-O-β-D-glucopyranosyloxy-1-hydroxy-6-(E)-tetradecene-8,10,12-triyne, were consistently isolated in ethyl acetate and n-butanol extracts (Yan et al., 2022; Chang et al., 2005). These compounds are known for their immunomodulatory and cytotoxic properties, and their recurrent detection suggests they may serve as chemotaxonomic markers or lead molecules for pharmaceutical development.

In parallel, Gas Chromatography–Mass Spectrometry (GC-MS) analyses were pivotal in identifying lipophilic and volatile compounds, including palmitic acid, oleic acid, α-pinene, phytol, and other terpenoids (Goudoum et al., 2016; Abiodun et al., 2020; Falowo et al., 2017; Quaglio et al., 2020). These molecules, often associated with anti-inflammatory and antimicrobial effects, are key to understanding the broader pharmacological effects of *B. pilosa*, especially in topical and nutraceutical applications.

The consistency in identification of certain compounds across studies, such as quercetin, rutin, isookanin, and glucuronylated quercetin (Fotso et al., 2014; Xin et al., 2021; Santos Filho et al., 2018), adds to the credibility of these findings. These flavonoids are among the most pharmacologically relevant, with a wide range of reported activities including enzyme inhibition, reactive oxygen species scavenging, and modulation of intracellular signaling pathways. Their ubiquity in *B. pilosa* across various extraction methods and geographic sources strengthens the case for their use as quality control markers in future standardization protocols.

Specifically, we identified 35 key metabolites with proven pharmacological relevance in *B. Pilosa*. These compounds were relevant particularly in modulating oxidative stress, lipid peroxidation, and inflammation, which are drivers of various diseases, including neurodegenerative and non-communicable diseases. Key phenolic acids like caffeic acid, gallic acid, ferulic acid, and chlorogenic acid (for example, 1-caffeoylquinic acid and 5Z-caffeoylquinic acid) exhibit antioxidant and anti-inflammatory properties by suppressing ROS generation, NF-κB activation, and pro-inflammatory cytokines such as TNF-α and IL-6. Flavonoids, including quercetin, kaempferol, rutin, isoquercetin, catechin, and luteolin derivatives, enhance cellular defenses through Nrf2 pathway activation, PI3K/Akt signaling, and membrane stabilization, mitigating neuroinflammation and mitochondrial dysfunction. Fatty acids like oleic acid, palmitic acid, and linoleic acid, with their esters (such as ethyl linoleate), support membrane integrity and exhibit anti-inflammatory and hypolipidemic effects. Terpenoids such as phytol, beta-caryophyllene, limonene, alpha-pinene, cadinene, and delta-cadinene contribute to antimicrobial, analgesic, and anti-inflammatory actions. Phytosterols like stigmasterol and beta-sitosterol have cholesterol-lowering and anti-diabetic properties, while tannic acid and vanillin exert broad-spectrum antioxidant and neuroprotective roles. Notably, paclitaxel, though rare in plants outside the *Taxus* genus, suggests a possible antitumor synergy within *B. pilosa*. Collectively, the phytocompounds in *B. pilosa* demonstrate a strong potential for integrative therapeutic strategies targeting oxidative damage, inflammation, and chronic degenerative diseases.

The presence of these diverse and bioactive metabolites aligns with findings from previous phytochemical reviews of *B. pilosa* and related *Asteraceae* species (Pegoraro et al., 2018; Pereira et al., 1999). The current synthesis, however, benefits from recent technological advancements in mass spectrometry and chromatography, allowing for a more comprehensive phytochemical fingerprint. Furthermore, the correlation between specific compounds (e.g., chlorogenic acid, rutin, phytol) and pharmacological outcomes such as antioxidant and anti-inflammatory effects reinforces the mechanistic plausibility of *B. pilosa*’s traditional uses in managing inflammatory and oxidative stress-related conditions.

### 4.4 Combined antioxidant and lipid peroxidation reducing potential of *Bidens pilosa*

According to the studies included, *Bidens pilosa* possesses a strong and varied antioxidant ability, working by both trapping radicals and influencing the body’s methods of protection. A number of extracts from *B. pilosa*, for example methanolic, aqueous and tea, have been shown to have strong free radical-scavenging effects in tests such as DPPH, ABTS and FRAP, proving that they can block reactive oxygen species and protect against oxidative damage (Akula and Odhav, 2008; Mohamed et al., 2024; Nxumalo et al., 2023; Phiri et al., 2024). Significant decreases in malondialdehyde (MDA) levels which indicate lipid peroxidation, are also observed in both laboratory and animal experiments (Abiodun et al., 2020; de Ávila et al., 2015; Bastos et al., 2016; Santos Filho et al., 2018). Lipid peroxidation plays a significant role in systemic inflammatory diseases, cardiovascular conditions, and metabolic disorders (Moretti et al., 2024; Wazir et al., 2023). Damage to cell membranes caused by oxidative stress can trigger immune responses, contribute to chronic inflammation, and exacerbate conditions such as atherosclerosis, diabetes, and autoimmune diseases (Bhol et al., 2024; Sadiq, 2021). Studies suggest that *Bidens pilosa*, a plant rich in flavonoids and phenolic acids, may offer protective benefits by reducing oxidative stress and inflammation (Varadhan et al., 2022). Through its antioxidant and anti-inflammatory properties, *Bidens pilosa* may help maintain cellular integrity, protect against membrane destabilization, and support overall physiological function (Aborode et al., 2022; Alqahtani et al., 2023; Tchekalarova and Tzoneva, 2023; Teleanu et al., 2022). The ability of *B. pilosa* to reduce MDA is important because lipid peroxidation is a main cause of cell membrane damage in diseases such as colitis and mucositis.

As well as direct radical removal, *B. pilosa* also activates the body’s internal antioxidant systems. Several studies show that in rodents and plants, extracts can boost the activity of SOD, CAT, POD and GSH (Tesfay et al., 2016; Mohamed et al., 2024; Abiodun et al., 2020). Because these enzymes are the first line of defense against oxidative stress, their increased levels by *B. pilosa* suggest both quick and lasting protection against redox imbalances. Removing radicals and supporting the body’s defense system makes the therapeutic potential of this plant more promising.

Importantly, these antioxidant activities are strongly associated with the phytochemical richness of *B. pilosa*, particularly its high total phenolic and flavonoid contents. Studies have consistently reported that flavonoids such as quercetin and phenolic acids like chlorogenic and caffeic acids contribute to its antioxidant effects by donating hydrogen atoms, chelating pro-oxidant metal ions, and modulating redox-sensitive signaling pathways (Yuniastri et al., 2022; Goudoum et al., 2016; Shandukani et al., 2018; Phiri et al., 2024). Through metal ion chelation, flavonoids including quercetin and kaempferol neutralize free radicals and prevent oxidative damage (Gulcin and Alwasel, 2022). By contributing hydrogen atoms to combat reactive oxygen species (ROS), phenolic acids, such as caffeic acid and chlorogenic acid, demonstrate strong radical-scavenging action (Mashinini et al., 2023). Additionally, by modulating oxidative stress signaling pathways, terpenoids like β-sitosterol strengthen cellular antioxidant defenses (Dwivedi et al., 2023; Liu et al., 2013)

These mechanisms collectively contribute to its broad-spectrum cytoprotective benefits, especially in inflammatory and oxidative-stress-related conditions.

The therapeutic utility of *B. pilosa* is further validated by *in vivo* models. Notably, formulations such as FITOPROT and Ecobidens^®^ significantly attenuated oxidative stress and inflammatory markers in models of 5-fluorouracil-induced oral and intestinal mucositis, respectively (Bastos et al., 2016; de Ávila et al., 2015; Santos Filho et al., 2018). These findings highlight the practical relevance of *B. pilosa*-based formulations in mitigating chemotherapy-induced toxicity. Moreover, studies on *B. pilosa* tea demonstrated its effectiveness in reversing oxidative damage in rats fed a high-oxalate diet, including restoration of GSH levels and reduction in MDA concentrations (Mohamed et al., 2024).

Another noteworthy observation is that methanolic and acetonic extracts of *B. pilosa* have shown antioxidant activities comparable to, or even surpassing, those of synthetic antioxidants such as butylated hydroxytoluene (BHT) (Goudoum et al., 2016). This not only positions *B. pilosa* as a promising candidate for inclusion in nutraceutical and pharmaceutical formulations but also aligns with current trends favoring natural over synthetic antioxidant sources due to safety concerns.

Collectively, these findings affirm that *B. pilosa* possesses strong antioxidant potential mediated by both its phytochemical constituents and its ability to modulate oxidative stress pathways. Its versatile applications across disease models, dietary supplementation, and food preservation further underscore its therapeutic and preventive promise.

### 4.5 Anti-inflammatory potential of *Bidens pilosa*

Our review confirms that *Bidens pilosa* possesses potent anti-inflammatory properties, mediated by a broad range of bioactive compounds that influence multiple immunological and molecular pathways. The findings reported across diverse studies highlight the plant’s capacity to downregulate pro-inflammatory mediators while promoting anti-inflammatory responses, indicating its promising therapeutic potential in managing inflammatory conditions.

Notably, *B. pilosa* inhibits major pro-inflammatory cytokines such as TNF-α, IL-1β, IL-6, and IL-8, while concurrently upregulating IL-10, a critical anti-inflammatory cytokine (Bastos et al., 2016; Quaglio et al., 2020). These effects suggest that the plant may restore immune homeostasis, particularly in pathological states marked by cytokine imbalances. The suppression of inducible nitric oxide synthase (iNOS) and cyclooxygenase-2 (COX-2) by isookanin, leading to decreased nitric oxide and prostaglandin E2 levels, further affirms the mechanistic depth of *B. pilosa*’s anti-inflammatory action (Xin et al., 2021). These outcomes are consistent with those of Caporali et al. (2022) and Chiang et al. (2004), who previously demonstrated that flavonoids in medicinal plants inhibit these same enzymes, key drivers of inflammation.

Additionally, *B. pilosa* modulates non-cytokine pathways involved in inflammation. It reduces histamine release from mast cells (Horiuchi and Seyama, 2008), inhibits the 5-lipoxygenase pathway (Akula and Odhav, 2008), and suppresses lymphocyte proliferation (Pereira et al., 1999), suggesting both early-phase and chronic inflammatory processes are targeted. These findings resonate with those by Tahya et al. (2023), who identified flavonoid glycosides in *B. pilosa* capable of regulating early immunologic responses.

Evidence from cellular models further strengthens these conclusions. Extracts from *B. pilosa* significantly inhibited IL-6 and nitric oxide production, downregulated NF-κB activation, and reduced myeloperoxidase (MPO) activity in macrophages and colon cell lines (Yan et al., 2022; Abiodun et al., 2020; Mohammed et al., 2024). These molecular effects suggest interference with canonical inflammatory signaling pathways such as NF-κB and MAPK, which are known to orchestrate a cascade of pro-inflammatory gene transcription (Wang et al., 2023; Detka et al., 2024). Importantly, the reduction in MPO activity and leukocyte infiltration in colitis models aligns with the anti-colitic effects previously reported by Abiodun et al. (2020) and Quaglio et al. (2020).

Animal studies provided compelling *in vivo* validation of these cellular findings. For instance, ethanol and aqueous extracts of *B. pilosa* significantly reduced paw edema and tissue inflammation in acute and chronic models (Fotso et al., 2014; Chih et al., 1995), while also mitigating cytokine expression in intestinal and renal tissues (Yang et al., 2018; Mohammed et al., 2024). These observations parallel those of Rodríguez Mesa et al. (2023), who reported that stigmasterol and friedelan-3-one in *B. pilosa* effectively attenuate inflammation through both COX-2 inhibition and antioxidant mechanisms.

Interestingly, the anti-inflammatory outcomes appear to be tissue-specific in certain delivery systems. For instance, FITOPROT-based mucoadhesive gels provided localized anti-inflammatory benefits without altering systemic cytokine profiles (Santos Filho et al., 2018). This property may be advantageous in the targeted treatment of oral and mucosal inflammations, as further demonstrated by Arantes et al. (2021b) in oral carcinoma and salivary epithelial models.

Despite these encouraging findings, the immunological effects of *B. pilosa* are not universally anti-inflammatory. Chang et al. (2005) reported that in certain conditions, the plant promotes a Th2-skewed immune response with elevated IgE levels, implying potential allergenic or pro-inflammatory consequences under specific immunological contexts. This duality underscores the importance of dosage, route of administration, and the physiological state of the host in mediating *B. pilosa*’s outcomes.

Collectively, these findings present *B. pilosa* as a multi-targeted anti-inflammatory agent with broad therapeutic applications. Its ability to modulate cytokine profiles, inflammatory enzymes, immune cell activity, and signaling pathways positions it as a strong candidate for managing inflammatory disorders such as colitis, arthritis, and mucosal inflammations. Given the increasing burden of chronic inflammatory diseases worldwide, especially in resource-limited settings, *B. pilosa* could be a cost-effective, plant-based therapeutic option.

Furthermore, its actions on oxidative and inflammatory pathways suggest potential for use in neuroinflammatory and metabolic conditions where similar mechanisms are implicated (Iqbal et al., 2025; Schreiner and Popescu, 2022). These findings also justify a deeper investigation into its role in modulating inflammasome activation, especially in diseases like Alzheimer’s and diabetes, where NLRP3 and oxidative stress play central roles (Qin et al., 2024; Alqahtani et al., 2023).

### 4.6 Limitations and future perspective

Although *Bidens pilosa* has many pharmacological uses, several obstacles prevent it from being used in clinical practice. Since there is a wide variety in what parts of plants are used, the solvents chosen, and the methods applied, it is difficult to compare results between studies. Due to the inconsistency in phytochemical analysis and no standard ways to dose herbal products, it is difficult to connect a dose with a specific effect and to pinpoint the active compounds. Also, because information on how these drugs are processed in the body and tested in humans is limited, it is difficult to apply findings to real clinical practice. The way phytochemicals work together to produce health benefits is not well understood. Despite these problems, the positive results from our systematic review highlight that more research is needed on *Bidens pilosa* to include it in health-promoting programs dealing with tissue toxicity, oxidative stress, lipid peroxidation, and inflammation.

To enhance a better use of *B. pilosa*, future studies should standardize how it is extracted and should test and measure all important active compounds in the plant. Bioassay-guided fractionation and using omics methods (metabolomics, transcriptomics and network pharmacology) can help find the targets and explain how they work. Evaluating the pharmacokinetics and toxicology of polyacetylenes and quercetin derivatives in major metabolites is essential. In particular, clinical trials that compare a treatment to a placebo and use standardized formulations should be given priority for conditions linked to oxidative stress and inflammation. Advanced computer models and systems biology methods should be used to study how various treatments work together and how to improve their results.

## 5 Conclusion

The review shows that *Bidens pilosa* is a medicinal plant with strong antioxidant and anti-inflammatory properties, confirmed by several *in vitro* and *in vivo* studies. The multiple metabolites in it, such as flavonoids, phenolic acids, polyacetylenes, and fatty acids, affect various biological functions and seem safe in animal studies. To make the most of its therapeutic benefits, people from different fields should collaborate to create standard ways, explore how it works, and confirm its safety and effectiveness in important clinical studies. With all these discoveries, *B. pilosa* has a strong chance to help develop useful plant-based treatments for diseases caused by oxidative stress, lipid peroxidation, and inflammation.

According to the findings of this systematic review, the following recommendations are presented to help direct future studies and the development of *Bidens pilosa* as a standardized phytotherapeutic agent:

i) Standardization of Extracts and Fractions of *B. pilosa* plant parts: Creating standard, detailed procedures for extracting metabolites and active components, their proportions, the temperature, the time involved, and the quantity of phytochemicals yielded will enable easy comparison of outcomes and ensure reproducibility.
ii) Separating the most active metabolites: Working on isolating and structurally defining the main bioactive compounds such as flavonoids, phenolic acids, and polyacetylenes, by using HPLC, LC-MS/MS, and NMR will allow better targeting of pharmacological experiments and improvement of compounds.
iii) Performing *in silico* pharmacokinetic (ADME) and toxicological predictions: Predicting whether key metabolites are bioavailable, safe, and can be properly formulated using Lipinski’s Rule of Five and SwissADME will facilitate the drug discovery and design process.
iv) Employing Molecular Docking and Molecular Dynamic Simulation Approaches: Molecular docking can be used to predict how *B. pilosa* compounds interact with proteins related to oxidative stress, inflammation, and metabolic disorders (such as COX-2, TNF-α, Nrf2, MAPKs). In addition, molecular dynamics simulations should be carried out to understand how strongly the ligand binds, how its structure is affected, and how the interaction with the protein changes over time.
v) Performing long-term and disease-specific research: By carrying out long-term *in vivo* experiments with tested animal models, the long-term effects of *B. pilosa’s* metabolites can be revealed. These studies should target inflammation using LPS-induced systemic inflammation and not only carrageenan-induced paw edema models. Models of metabolic disorders, diabetes, neurodegeneration, and cancer should also be used, along with testing antiproliferative and cytotoxic effects on both human tumor xenografts and cancer cell cultures, *in vitro.*

## Data Availability

The original contributions presented in the study are included in the article/Supplementary Material, further inquiries can be directed to the corresponding author.

## Abbreviations

*Bidens pilosa*, *Bidens pilosa*; SYRCLE, Systematic Review Centre for Laboratory Animal Experimentation; PRISMA, Preferred Reporting Items for Systematic Reviews and Meta-Analyses; RoB, – Risk of Bias; SciRAP, Science in Risk Assessment and Policy; TPC, Total Phenolic Content; TFC, Total Flavonoid Content; TTC, Total Tannin Content; TSC, Total Saponin Content; UV Spec, Ultraviolet Spectrophotometry; GC-MS, Gas Chromatography-Mass Spectrometry; HPLC, High-Performance Liquid Chromatography; LC-MS/MS, Liquid Chromatography-Tandem Mass Spectrometry; LC-HR-MS, Liquid Chromatography-High-Resolution Mass Spectrometry; NA, Not Applicable; CID, Compound Identifier.
