# Supplementary material for: Exploring the phytochemical profile, antioxidant and anti-inflammatory potential of Bidens pilosa: A Systematic Review
Source: Front Pharmacol. 2025 Aug 1;16:1569527. doi: 10.3389/fphar.2025.1569527 (PMC12355053; doi:10.3389/fphar.2025.1569527)
Supplement: Supplementary file 1 [file Table1.docx]

**Supplementary Table 1: Phytochemical Analysis of *Bidens pilosa* Extracts and Phytocompounds Identified**

| **S/N** | **Extract** | **UV-Spec** | **GC-MS** | **HPLC** | **LC-MS/MS** | **LC-HR-MS** | **Reference** |
| --- | --- | --- | --- | --- | --- | --- | --- |
| 1 | Glycolic Extract | NA | NA | NA | NA | NA | (Bastos et al., 2016) |
| 2 | Ethyl acetate fractionation. | NA | NA | **Flavonoids**: quercetin and isookanin | NA | NA | (Fotso et al., 2014) |
| 3 | Decoctation | NA | NA | NA | NA | NA | (Chih et al., 1995) |
| 4 | Ethanol-water, petroleum ether, dichloromethane, ethyl acetate, and n-butanol. | NA | NA | polyacetylenic glucosides (4-O-β-D-glucopyranosyloxy-1-hydroxy-6-(E)-tetradecene-8,10,12-triyne; 3-O-β-D-glucopyranosyloxy-1-hydroxy-6-(E)-tetradecene-8,10,12-triyne; 2-O-β-D-glucopyranosyloxy-1-hydroxy-5-(E)-tetradecene-7,9,11-triyne; Ichthyothereol acetate) | NA | polyacetylenic glucosides, (4-O-β-D-glucopyranosyloxy-1-hydroxy-6-(E)-tetradecene-8,10,12-triyne) | (Yan et al., 2022) |
| 5 | Mthyl acetate (EtOAc), and n-butanol (n-BuOH) | NA | NA | **NA** | Isookanin and m/z 269 [M−H−H₂O] | Isookanin | (Xin et al., 2021) |
| 6 | Methanolic extraction | NA | NA | To isolate the polyacetylene PA-1. PA-1 is identified as a polyacetylene compound, 2-O-β-D-glucosyltrideca-11E-en-3,5,7,9-tetrayn-1,2-diol | **NA** | NA | (Pereira et al., 1999) |
| 7 | Carboxy-metthyl-cellulose sodium (CMC-Na) | NA | NA | Caffeic acid, Caffeoylquinic acid, Hyperin and Isoquercitrin | NA | NA | (Horiuchi & Seyama, 2008) |
| 8 | CO₂ extraction, Chloroform, n-hexane, hydroalcoholic (ethanol-water 1:1), ethyl acetate | NA | GC-MS analysis identified compounds such as palmitic acid (30%), oleic acid (27%), linoleic acid (24.3%), linolenic acid (3.8%), phytol (0.139%**)**, along with traces of alkanes, fatty acid ethyl esters, and sterols | Tannins, Alkaloids, Steroids, Cardiac Glycosides Phenols and Terpenoids | NA | NA | (Quaglio et al., 2020) |
| 9 | Glycerinated extract: glycerol-based (hydroalcoholic) extraction | NA | NA | NA | NA | NA | (Arantes et al., 2021) |
| 10 | n-butanol | NA | NA | polyacetylenic glucosides (2-β-D-glucopyranosyloxy-1-hydroxy-5(E)-tridecene-7,9,11-triyne and 3-β-D-glucopyranosyloxy-1-hydroxy-6(E)-tetradecene-8,10,12-triyne) | NA | NA | (Chang et al., 2005) |
| 11 | Ethanol, n-butanol and ethyl acetate | NA | NA | 4-O-(2″-O-acetyl-6″-O-p-coumaroyl-β-D-glucopyranosyl)-p-coumaric acid (4-ACGC). The HPLC results confirmed its purity as not less than 98%. | NA | NA | (Y. Yang et al., 2018) |
| 12 | Decoctation | NA | NA | NA | NA | NA | (Pegoraro et al., 2018) |
| 13 | Glycolic Extract based polaxamer | NA | NA | Polyphenols | NA | NA | (de Ávila et al., 2015) |
| 14 | Glycolic extract | NA | NA | NA | Flavonoids:Rutin, Glucuronylated quercetin, Dimethylquercetin rutenoside | NA | (Santos Filho et al., 2018) |
| 15 | Aqueous extraction | The UV Spectroscopy analysis is applied for assessing total phenolic content (TPC) and total flavonoid content (TFC) | NA | NA | NA | NA | (Mohamed et al., 2024) |
| 16 | Methanolic and Aqueous | TPC (quantified in gallic acid equivalents), *Bidens pilosa* extracts were among the top 5 plants with high phenolic content. | NA | NA | NA | NA | (Akula & Odhav, 2008) |
| 17 | Methanol extract | NA | NA | Soluble carbohydrates (raffinose, glucose, fructose, sucrose | NA | NA | (Tesfay et al., 2016) |
| 18 | ethanol and aqueous extraction | TPC (quantified in phenol equivalents) | secondary metabolites like terpenoids, steroids, tannins, and flavonoids. | NA | NA | NA | (Yuniastri et al., 2022) |
| 19 | Methanol extract | NA | 2-Undecenal, (43.93% abundance),O-Methylisourea (13.20%), Hexadecanoic acid, methyl ester (6.94%), Oleic acid (6.19%), 9-Octadecenoic acid (Z)-methyl ester (5.29%), Methyl [2-(1-methylpyrazol-4-yl)ethyl]amine (4.11%), Hexadecanal (3.72%), Methyl stearate (2.48%), n-Hexadecanoic acid (2.00%) | NA | NA | NA | (Abiodun et al., 2020) |
| 20 | Methanolic extract | NA | NA | NA | identified numerous compounds such as phenolic acids, organic acids, flavonoids, ellagitannins, monosaccharides, and unknown polyphenols across the plant extracts | LC-HR-MS analysis was used for profiling a wide range of bioactive compounds such as phenolic acids, flavonoids, ellagitannins, and other polyphenols. | (Nxumalo et al., 2023) |
| 21 | Hexane, dichloromethane, ethyl acetate, acetone, and methanol | NA | NA | NA | NA | NA | (Shandukani et al., 2018) |
| 22 | Hydrodistillation | NA | The GC-MS analysis identified 27 compounds, accounting for 97.57% of the total oil content. Major compounds include: α-Pinene (14.7%), Caryophyllene (13.5%), β-Ocimene (12.8%), Cadinene (10.1%) | NA | NA | NA | (Goudoum et al., 2016) |
| 23 | Acetone extract | NA | NA | NA | LC-MS/MS was used to identify the protein present in the acetonic fraction of Bidens pilosa. This was characterized as a novel lectin, maturase K. | NA | (Mota et al., 2019) |
| 24 | Methanol and ethanol | Total Phenolic Content (TPC) and Total Flavonoid Content (TFC) at specific wavelengths (745 nm and 429 nm respectively). | NA | NA | NA | NA | (Phiri et al., 2024) |
| 25 | Aqueous, methanol, acetone, and ethyl acetate | NA | NA | The HPLC analysis identified and quantified phenolic acids (e.g., gallic acid, chlorogenic acid, caffeic acid) and flavonoids (e.g., quercetin, rutin) present in the extracts. | NA | NA | (Nguyen et al., 2023) |
| 26 | DMSO (Dimethyl Sulfoxide) extract | NA | GC-MS analysis in external studies of Bidens pilosa, showing flavonoids and other phenolic | NA | NA | NA | (Said et al., 2024) |
| 27 | Methanol extract | This is used to analyze the scavenging properties of phenolic compounds. | NA | Specific phenolics identified include caftaric acid, and rutin | LC-MS/MS revealed various compounds, including oligosaccharides, disaccharides, fatty acids, monoglycerides, peptides, diterpenoids, and amino acids. Different plant parts showed distinct distributions of these metabolites. | LC-HR-MS analysis helped annotate compounds with high accuracy, identifying diverse secondary metabolites such as flavonoids, terpenoids, phenylpropanoids, and others that influence the pharmacological effects of the plant. | (Angelini et al., 2021) |
| 28 | Aqueous extraction | NA | The GC-MS analysis identified various phenolic and phytochemical compounds such as menthol, β-myrcene, α-cadinol, eugenol, caffeine, phytol, and others from the aqueous extracts of the Thai weeds. | NA | NA | NA | (U-Yatung et al., 2020) |
| 29 | Ethanol extractions, Ethanolic extraction and Methanolic extracts | extracts were analyzed the level of phenolic and flavonoid via UV-spectrophotometry | NA | NA | NA | NA | (Oduntan et al., 2018) |
| 30 | Ethanol extract | UV Spectroscopy in this study was used to measure the content of total chlorophyll (a and b) and total carotenoids in the extracts | GC-MS identified volatile compounds such as tetradecanoic acid, n-hexadecanoic acid, hexadecanoic acid ethyl ester, phytol, linoleic acid ethyl ester, DL-alpha-tocopherol, phenol *Bidens pilosa* extracts. | NA | NA | NA | (Falowo et al., 2017) |
| 31 | Methanolic extraction | UV/Vis spectrophotometer usage for phenolic and flavonoids content determination | Six bioactive volatile compounds were identified in the methanolic leaves extract using GC-MS, such as:1,3,6,10-Dodecatetraene, 3,7,11-trimethyl-(Z,E); 1H-3A, 7-Methanozulene, Octahydro-1,4,9,9-tetramethyl; 9H–Fluorene, 9-Diazo; 1-Octadecyne; N-Hexadecanoic acid; 3,7,11,15-Tetramethyl-2-Hexadecen-1-ol | NA | It was used to quantified bioactive compounds, specifically phenolics (catechin, kaempferol, ferulic acid, gallic acid) and anticancerous compound paclitaxel | NA | (Singh, Passsari, Singh, Leo, Subbarayan, Kumar, Singh, lalhlenmawia, et al., 2017) |
| 32 | Ethanolic extract | UV Spectroscopy in the for analyzing phytochemicals like flavonoids and phenolic acids | NA | HPLC analysis in the article is used to identify phenolic acids, flavonoids, and their derivatives. | The LC-MS/MS analysis identified a broad range of phytochemicals, including phenolic acids, flavonoids, coumarins, and fatty acids | High-resolution mass spectrometry (LC-HR-MS) was employed to characterize 137 phytochemicals, such as hydroxybenzoic acids, coumarin derivatives, and caffeoylquinic acid derivatives. | (Idris et al., 2023) |
| 33 | Decoction | NA | NA | NA | NA | NA | (Memon et al., 2020) |
| 34 | Ethanol extraction | NA | NA | HPLC, the study identifies and quantifies polyphenols and flavonoids, mentioning total polyphenol content as 107.49 ± 4.04 mg GAE/g and total flavonoid content as 165.63 ± 2.90 mg QE/g extract. | NA | NA | (Son et al., 2022) |
| 35 | Methanol, ethanol, acetone, hexane, and Aqueous | The UV-Visible spectrophotometer was used for phenolic compounds as part of the total phenolic content determination. | NA | The study identified phenolic compounds through High-Performance Liquid Chromatography (HPLC). These compounds included gallic acid, tannic acid, vanillin, and other similar polyphenolic constituents, aligning with the plant's phytochemical profile. | NA | NA | (Mashinini et al., 2023) |
| 36 | Ethanol or glycerin extraction | NA | NA | HPLC is used for the chemoprofile evaluation, identifying and quantifying active compounds such as rutin, glucuronidated quercetin, and dimethyl quercetin rutinoside) found in the Bidens pilosa extract. | NA | NA | (Arantes et al., 2021) |
| 37 | Diethyl ether | UV–Vis spectrophotometer to evaluate antioxidant activity through methods such as DPPH free radical scavenging | GC-MS analysis identified terpenes as the major components in the essential oils of *Bidens pilosa*. include: β-caryophyllene (10.9% in leaves, 5.1% in flowers); δ-cadinene (7.82% in leaves, 6.13% in flowers); Other terpenes like α-pinene, limonene, and caryophyllene oxide. | NA | NA | NA | (Deba et al., 2007) |
| 38 | Ethanol, ethyl acetate (EA), n-butanol (BuOH) and aqueous | UV spectroscopy was employed | NA | HPLC profiling revealed several phenolic compounds in the BuOH fraction, including quercetin 3-O-rabinobioside, quercetin 3-O-rutinoside, chlorogenic acid, 3,4-di-O-caffeoylquinic acid, 3,5-di-O-caffeoylquinic acid, 4,5-di-O-caffeoylquinic acid, jacein, and centaurein. | LC-MS analysis was performed for the metabolite profiling of the BuOH fraction. Major compounds included quercetin derivatives, caffeoylquinic acids, and others. Specific pseudomolecular weights were obtained to characterize these compounds. | NA | (Chiang et al., 2004) |
| 39 | Petroleum ether, ethyl acetate, n-BuOH, and aqueous fractions. | NA | NA | NA | NA | NA | (Yi et al., 2016) |
| 40 | NA (should be excluded) | NA | NA | NA | NA | NA | (Liu et al., 2013) |
| 41 | Methanolic | NA | NA | NA | NA | NA | (Muchuweti et al., 2007) |
| 42 | Hydroethanol maceration | The UV-Vis spectra were obtained to characterize the compounds. | NA | HPLC identified the main compound as quercetin 3,3′-dimethyl ether 7-O-β-D-glycopyranoside, a flavonoid with significant antioxidant properties. | NA | NA | (Kviecinski et al., 2011) |
| 43 | Methanol, Cyclohexane, and Methylene chloride extracts | NA | NA | HPLC is used to identify and separate various non-volatile compounds. It identifies flavonoids, including rutin and six caffeic acid derivatives, were analyzed using HPLC. | NA | NA | (Horiuchi et al., 2010) |
| 44 | Methanolic extracts | UV spectroscopy was used indirectly to measure absorbance for assessing total phenolic content and antioxidant activities. | NA | The phenolic acids identified via HPLC include gallic acid, protochatechuic acid, hydroxybenzoic acid, catechin, caffeic acid, vanillic acid, coumaric acid, and ferullic acid. | NA | NA | (Chipurura et al., 2013) |
| 45 | Acetone, methanol, and Aqueous extractions | UV spectroscopy was utilized to measure the total phenolic content, flavonoids, flavanols, and proanthocyanidins. The compounds identified in the extracts (e.g., tannic acid, quercetin equivalents) suggest the presence of polyphenolic compounds. | NA | NA | NA | NA | (Adedapo, 2011) |
| 46 | Ethanolic extract | The UV Spectroscopy analysis quantifies the total flavonoid content, which is calculated as rutin | NA | NA | NA | NA | (Yuan et al., 2008) |
| 47 | Ethanolic extraction | NA | NA | NA | NA | NA | (Cortés-Rojas et al., 2011) |
| 48 | Petroleum ether, ethyl acetate, n-butanol, and Aqueous | NA | NA | In the HPLC analysis, the active flavonoid compound BP-6 (5,7,4'-trihydroxy-3,3'-dimethylflavonol) was isolated and identified. Its purity was above 99%, with a detected content of 0.15% in the EE-BP fraction. | NA | NA | (Wu et al., 2012) |
| 49 | Ethanol (EtOH) and Ethylacetate/ethanol (EA/EtOH) extraction | NA | NA | HPLC profiling identifies several major compounds, including: 3,4-di-O-caffeoylquinic acid; 3,5-di-O-caffeoylquinic acid; 4,5-di-O-caffeoylquinic acid;Ethyl caffeat | NA | NA | (H. L. Yang et al., 2006) |
| 50 | Petroleum ether | NA | Stigmasterol, Beta-Sitosterol, and Friedelan-3-one,Ethyl Linoleate, Ethyl Linolenate , Neophytadiene,Dihydroactinidiolide, Tetrapentacontane, and Methyl Commate C., | NA | NA | NA | (Melissa et al., 2023) |

**NA=** Not Applicable**, TPC =** Total Phenolic Content**, TFC =** Total Flavonoid Content**, TTC =** Total Tannin Content, **TSC =** Total Saponin Content, **UV Spec =** Ultraviolet Spectrophotometry**, GC-MS =** Gas Chromatography-Mass Spectrometry, **HPLC =** High-Performance Liquid Chromatography, **LC-MS/MS =** Liquid Chromatography-Tandem Mass Spectrometry, **LC-HR-MS =** Liquid chromatography-high-resolution mass spectrometry.

**References**

Abiodun, O. O., Sosanya, A. S., Nwadike, N., & Oshinloye, A. O. (2020). Beneficial effect of Bidens pilosa L. (Asteraceae) in a rat model of colitis. *Journal of Basic and Clinical Physiology and Pharmacology*, *31*(6), 1–9. https://doi.org/10.1515/jbcpp-2019-0166

Adeolu Adedapo, F. J. and A. A. (2011). *Comparison of the nutritive value and biological activities of the acetone, methanol and water extracts of the leaves of Bidens pilosa and Chenopodium album - PubMed*. Acta Poloniae Pharmaceutica ñ Drug Research, Vol. 68 No. 1 Pp. 83ñ92, 2011.

Akula, U. S., & Odhav, B. (2008). *In vitro 5-Lipoxygenase inhibition of polyphenolic antioxidants from undomesticated plants of South Africa*. *2*(9), 207–212.

Angelini, P., Matei, F., Flores, G. A., Pellegrino, R. M., Vuguziga, L., Venanzoni, R., Tirillini, B., Emiliani, C., Orlando, G., Menghini, L., & Ferrante, C. (2021). Metabolomic Profiling, Antioxidant and Antimicrobial Activity of Bidens pilosa. *Processes 2021, Vol. 9, Page 903*, *9*(6), 903. https://doi.org/10.3390/PR9060903

Arantes, D. A. C., da Silva, A. C. G., Freitas, N. M. A., Lima, E. M., de Oliveira, A. C., Marreto, R. N., Mendonça, E. F., & Valadares, M. C. (2021). Safety and efficacy of a mucoadhesive phytomedication containing curcuminoids and Bidens pilosa L. extract in the prevention and treatment of radiochemotherapy-induced oral mucositis: Triple-blind, randomized, placebo-controlled, clinical trial. *Head and Neck*, *43*(12), 3922–3934. https://doi.org/10.1002/HED.26892;PAGEGROUP:STRING:PUBLICATION

Arantes, D. A. C., da Silva, A. C. G., Lima, E. M., Alonso, E. C. P., Marreto, R. N., Mendonça, E. F., Valadares, M. C., & Batista, A. C. (2021). Biological effects of formulation containing curcuminoids and *Bidens Pilosa L*. in oral carcinoma cell line. *Brazilian Oral Research*, *35*, e063. https://doi.org/10.1590/1807-3107BOR-2021.VOL35.0063

Bastos, C. C. C., Ávila, P. H. M. de, Filho, E. X. dos S., Ávila, R. I. de, Batista, A. C., Fonseca, S. G., Lima, E. M., Marreto, R. N., Mendonça, E. F. de, & Valadares, M. C. (2016). Use of Bidens pilosa L. (Asteraceae) and Curcuma longa L. (Zingiberaceae) to treat intestinal mucositis in mice: Toxico-pharmacological evaluations. *Toxicology Reports*, *3*, 279–287. https://doi.org/10.1016/j.toxrep.2015.10.013

Chang, C. L. T., Kuo, H. K., Chang, S. L., Chiang, Y. M., Lee, T. H., Wu, W. M., Shyur, L. F., & Yang, W. C. (2005). The distinct effects of a butanol fraction of Bidens pilosa plant extract on the development of Th1-mediated diabetes and Th2-mediated airway inflammation in mice. *Journal of Biomedical Science*, *12*(1), 79–89. https://doi.org/10.1007/S11373-004-8172-X/METRICS

Chiang, Y. M., Chuang, D. Y., Wang, S. Y., Kuo, Y. H., Tsai, P. W., & Shyur, L. F. (2004). Metabolite profiling and chemopreventive bioactivity of plant extracts from Bidens pilosa. *Journal of Ethnopharmacology*, *95*(2–3), 409–419. https://doi.org/10.1016/J.JEP.2004.08.010

Chih, H. W., Lin, C. C., & Tang, K. S. (1995). Anti-inflammatory activity of Taiwan folk medicine “ham-hong-chho” in rats. *The American Journal of Chinese Medicine*, *23*(3–4), 273–278. https://doi.org/10.1142/S0192415X95000328;ISSUE:ISSUE:10.1142/AJCM.23.ISSUE-03N04;PAGEGROUP:STRING:PUBLICATION

Chipurura, B., Muchuweti, M., & Bhebhe, M. (2013). An assessment of the phenolic content, composition and antioxidant capacity of selected indigenous vegetables of Zimbabwe. *Acta Horticulturae*, *979*, 611–620. https://doi.org/10.17660/ACTAHORTIC.2013.979.66

Cortés-Rojas, D. F., Souza, C. R. F., & Oliveira, W. P. (2011). Optimisation of the extraction of phenolic compounds and antioxidant activity from aerial parts of Bidens pilosa L. using response surface methodology. *International Journal of Food Science and Technology*, *46*(11), 2420–2427. https://doi.org/10.1111/J.1365-2621.2011.02765.X

de Ávila, P. H. M., de Ávila, R. I., dos Santos Filho, E. X., Cunha Bastos, C. C., Batista, A. C., Mendonça, E. F., Serpa, R. C., Marreto, R. N., da Cruz, A. F., Lima, E. M., & Valadares, M. C. (2015). Mucoadhesive formulation of Bidens pilosa L. (Asteraceae) reduces intestinal injury from 5-fluorouracil-induced mucositis in mice. *Toxicology Reports*, *2*, 563. https://doi.org/10.1016/J.TOXREP.2015.03.003

Deba, F., Xuan, T. D., Yasuda, M., & Tawata, S. (2007). Chemical composition and antioxidant, antibacterial and antifungal activities of the essential oils from Bidens pilosa Linn. var. Radiata. *Food Control*, *19*(4), 346–352. https://doi.org/10.1016/J.FOODCONT.2007.04.011

Falowo, A. B., Muchenje, V., Hugo, A., Aiyegoro, O. A., & Fayemi, P. O. (2017). Antioxidant activities of Moringa oleifera L. and Bidens pilosa L. leaf extracts and their effects on oxidative stability of ground raw beef during refrigeration storage. *CyTA - Journal of Food*, *15*(2), 249–256. https://doi.org/10.1080/19476337.2016.1243587

Fotso, A. F., Longo, F., Djomeni, P. D. D., Kouam, S. F., Spiteller, M., Dongmo, A. B., & Savineau, J. P. (2014). Analgesic and antiinflammatory activities of the ethyl acetate fraction of Bidens pilosa (Asteraceae). *Inflammopharmacology*, *22*(2), 105–114. https://doi.org/10.1007/s10787-013-0196-2

Goudoum, A., Abdou, A. B., Ngamo, L. S. T., Ngassoum, M. B., & Mbofung, C. M. F. (2016). Antioxidant activities of essential oil of Bidens pilosa (Linn. Var. Radita) used for the preservation of food qualities in North Cameroon. *Food Science and Nutrition*, *4*(5), 671–678. https://doi.org/10.1002/FSN3.330

Horiuchi, M., & Seyama, Y. (2008). Improvement of the antiinflammatory and antiallergic activity of Bidens pilosa L. var. radiata SCHERFF treated with enzyme (Cellulosine). *Journal of Health Science*, *54*(3), 294–301. https://doi.org/10.1248/jhs.54.294

Horiuchi, M., Wachi, H., & Seyama, Y. (2010). Effects of Bidens pilosa L. var. radiata Scherff on experimental gastric lesion. *Journal of Natural Medicines*, *64*(4), 430–435. https://doi.org/10.1007/s11418-010-0426-5

Idris, O. A., Kerebba, N., Horn, S., Maboeta, M. S., & Pieters, R. (2023). Phytochemical-Based Evidence of the Health Benefits of Bidens Pilosa Extracts and Cytotoxicity. *Chemistry Africa*, *6*(4), 1767–1788. https://doi.org/10.1007/s42250-023-00626-2

Kim, S., & Bolton, E. E. (2023). PubChem: A Large-Scale Public Chemical Database for Drug Discovery. *Open Access Databases and Datasets for Drug Discovery*, 39–66. https://doi.org/10.1002/9783527830497.CH2

Kviecinski, M. R., Felipe, K. B., Correia, J. F. G., Ferreira, E. A., Rossi, M. H., Gatti, F. de M., Filho, D. W., & Pedrosa, R. C. (2011). Brazilian Bidens pilosa linné yields fraction containing quercetin-derived flavonoid with free radical scavenger activity and hepatoprotective effects. *Libyan Journal of Medicine*, *6*(1), 1–8. https://doi.org/10.3402/LJM.V6I0.5651

Liu, J. R., Ye, Y. L., Lin, T. Y., Wang, Y. W., & Peng, C. C. (2013). Effect of floral sources on the antioxidant, antimicrobial, and anti-inflammatory activities of honeys in Taiwan. *Food Chemistry*, *139*(1–4), 938–943. https://doi.org/10.1016/j.foodchem.2013.02.015

Mashinini, P. P., Chihomvu, P., Pillay, M., & Takaidza, S. (2023). Phytochemical analysis and anti-mycobacterium activity of Bidens pilosa crude extracts. *Journal of Biotech Research*, *15*(September), 116–137.

Melissa, X., Andres, L., Bolaños, C., Mej, A., Pombo, L. M., Costa, G. M., Paola, S., & Gonz, S. (2023). *Immunomodulatory Properties of Natural Extracts and Compounds Derived from Bidens pilosa L .: Literature Review*.

Memon, F. Q., Yang, Y., Lv, F., Soliman, A. M., Chen, Y., Sun, J., Wang, Y., Zhang, G., Li, Z., Xu, B., Gadahi, J. A., & Si, H. (2020). *Effects of probiotics and Bidens pilosa on the performance and gut health of chicken during induced E. tenella infection*. https://doi.org/10.21203/RS.3.RS-18318/V1

Mohamed, R. S., Ramadan, M. M., Fouda, K., Ghanem, K. Z., Omara, E. A., & Abdel-Aziz, S. A. (2024). Preventive Impacts of Black Tea, Green Tea and Bidens pilosa on Renal Stone Formation in Rats: Antioxidant and Anti-inflammatory Pathways. *Egyptian Journal of Chemistry*, *67*(12), 423–432. https://doi.org/10.21608/EJCHEM.2024.317701.10337

Mota, C. M., Santiago, F. M., Cardoso, M. de R. D., Rostkowska, C., de Oliveira, T. C., Silva, D. A. de O., Pirovani, C. P., Mineo, T. W. P., & Mineo, J. R. (2019). Acetonic fraction of Bidens pilosa enriched for maturase K is able to control cerebral parasite burden in mice experimentally infected with Toxoplasma gondii. *Frontiers in Veterinary Science*, *6*(MAR), 1–13. https://doi.org/10.3389/fvets.2019.00055

Muchuweti, M., Mupure, C., Ndhlala, A., Murenje, T., & Benhura, M. A. N. (2007). Screening of antioxidant and radical scavenging activity of Vigna ungiculata, Bidens pilosa and Cleome gynandra. *American Journal of Food Technology*, *2*(3), 161–168. https://doi.org/10.3923/AJFT.2007.161.168

Nguyen, T. H. D., Vu, D. C., Hanh, P. Q. P., Vo, X. T., Nguyen, V. C., Nguyen, T. N., Nguyen, L. L. P., & Baranyai, L. (2023). Comparative analysis of phenolic content and in vitro bioactivities of Bidens pilosa L. flowers and leaves as affected by extraction solvents. *Journal of Agriculture and Food Research*, *14*, 100879. https://doi.org/10.1016/J.JAFR.2023.100879

Nxumalo, K. A., Aremu, A. O., & Fawole, O. A. (2023). Metabolite profiling, antioxidant and antibacterial properties of four medicinal plants from Eswatini and their relevance in food preservation. *South African Journal of Botany*, *162*, 719–729. https://doi.org/10.1016/J.SAJB.2023.10.008

Oduntan, A. O., Fasoyiro, S. B., Akinfasoye, J. A., Adeboyejo, F. O., & Akintoye, H. A. (2018). Antioxidant and proximate properties of underutilized vegetables in western Nigeria. *Acta Horticulturae*, *1225*, 255–260. https://doi.org/10.17660/ACTAHORTIC.2018.1225.35

Pegoraro, C. M. R., Nai, G. A., Garcia, L. A., Serra, F. de M., Alves, J. A., Chagas, P. H. N., Oliveira, D. G. de, & Zocoler, M. A. (2018). Protective effects of Bidens pilosa on hepatoxicity and nephrotoxicity induced by carbon tetrachloride in rats. *Drug and Chemical Toxicology*, *44*(1), 64–74. https://doi.org/10.1080/01480545.2018.1526182;WGROUP:STRING:PUBLICATION

Pereira, R. L. C., Ibrahim, T., Lucchetti, L., Da Silva, A. J. R., & De Moraes, V. L. G. (1999). Immunosuppressive and anti-inflammatory effects of methanolic extract and the polyacetylene isolated from Bidens pilosa L. *Immunopharmacology*, *43*(1), 31–37. https://doi.org/10.1016/S0162-3109(99)00039-9

Phiri, H., Lumai, A., Zombe, K., & Nyirenda, J. (2024). Evaluation of antioxidant activity of selected wild fruits and vegetables from Zambia. *Food and Humanity*, *3*, 100390. https://doi.org/10.1016/J.FOOHUM.2024.100390

Quaglio, A. E. V., Cruz, V. M., Almeida-Junior, L. D., Costa, C. A. R. A., & Di Stasi, L. C. (2020). Bidens pilosa (Black Jack) Standardized Extract Ameliorates Acute TNBS-induced Intestinal Inflammation in Rats. *Planta Medica*, *86*(05), 319–330. https://doi.org/10.1055/A-1089-8342

Rodríguez Mesa, X. M., Contreras Bolaños, L. A., Modesti Costa, G., Mejia, A. L., & Santander González, S. P. (2023). A Bidens pilosa L. Non-Polar Extract Modulates the Polarization of Human Macrophages and Dendritic Cells into an Anti-Inflammatory Phenotype. *Molecules 2023, Vol. 28, Page 7094*, *28*(20), 7094. https://doi.org/10.3390/MOLECULES28207094

Said, W., Khattab, A. A., Hamed, S. A., Abo-Elmaaty, S. A., & Khalil, H. (2024). Identification of Bioactive and Anticancer Properties of Bidens Pilosa in-vitro Evidence. *Asian Pacific Journal of Cancer Prevention : APJCP*, *25*(10), 3551. https://doi.org/10.31557/APJCP.2024.25.10.3551

Santos Filho, E. X. dos, Arantes, D. A. C., Oton Leite, A. F., Batista, A. C., Mendonça, E. F. de, Marreto, R. N., Naves, L. N., Lima, E. M., & Valadares, M. C. (2018). Randomized clinical trial of a mucoadhesive formulation containing curcuminoids (Zingiberaceae) and Bidens pilosa Linn (Asteraceae) extract (FITOPROT) for prevention and treatment of oral mucositis - phase I study. *Chemico-Biological Interactions*, *291*, 228–236. https://doi.org/10.1016/J.CBI.2018.06.010

Shandukani, P. D., Tshidino, S. C., Masoko, P., & Moganedi, K. M. (2018). Antibacterial activity and in situ efficacy of Bidens pilosa Linn and Dichrostachys cinerea Wight et Arn extracts against common diarrhoea-causing waterborne bacteria. *BMC Complementary and Alternative Medicine*, *18*(1). https://doi.org/10.1186/S12906-018-2230-9

Singh, G., Passsari, A. K., Singh, P., Leo, V. V., Subbarayan, S., Kumar, B., Singh, B. P., & Kumar, N. S. (2017). *Pharmacological potential of Bidens pilosa L . and determination of bioactive compounds using UHPLC-QqQ LIT -MS / MS and GC / MS*. 1–16. https://doi.org/10.1186/s12906-017-2000-0

Singh, G., Passsari, A. K., Singh, P., Leo, V. V., Subbarayan, S., Kumar, B., Singh, B. P., lalhlenmawia, H., & Kumar, N. S. (2017). Pharmacological potential of Bidens pilosa L. and determination of bioactive compounds using UHPLC-QqQLIT-MS/MS and GC/MS. *BMC Complementary and Alternative Medicine*, *17*(1), 1–16. https://doi.org/10.1186/S12906-017-2000-0/TABLES/9

Son, N. H., Tuan, N. T., & Tran, T. M. (2022). Investigation of chemical composition and evaluation of antioxidant, antibacterial and antifungal activities of ethanol extract from *Bidens pilosa* L. *Food Science and Technology*, *42*, e22722. https://doi.org/10.1590/FST.22722

Tesfay, S. Z., Mathe, S., Modi, A. T., & Mabhaudhi, T. (2016). A Comparative Study on Antioxidant Potential of Selected African and Exotic Leafy Vegetables. *HortScience*, *51*(12), 1529–1536. https://doi.org/10.21273/HORTSCI11161-16

Tsuruta, K., Shidara, T., Miyagishi, H., Nango, H., Nakatani, Y., Suzuki, N., Amano, T., Suzuki, T., & Kosuge, Y. (2023). Anti-Inflammatory Effects of Miyako Bidens pilosa in a Mouse Model of Amyotrophic Lateral Sclerosis and Lipopolysaccharide-Stimulated BV-2 Microglia. *International Journal of Molecular Sciences 2023, Vol. 24, Page 13698*, *24*(18), 13698. https://doi.org/10.3390/IJMS241813698

U-Yatung, S., Suebsaiprom, W., Pornprom, T., & Chompoo, J. (2020). Performance of some thai weed extracts on antioxidants and atherosclerosis-related enzymes. *Agrivita*, *42*(2), 243–254. https://doi.org/10.17503/AGRIVITA.V0I0.2322

Wu, J., Wan, Z., Yi, J., Wu, Y., Peng, W., & Wu, J. (2012). Investigation of the extracts from Bidens pilosa Linn. var. radiata Sch. Bip. for antioxidant activities and cytotoxicity against human tumor cells. *Journal of Natural Medicines 2012 67:1*, *67*(1), 17–26. https://doi.org/10.1007/S11418-012-0639-X

Xin, Y.-J., Choi, S., Roh, K.-B., Cho, E., Ji, H., Weon, J. B., Park, D., Whang, W. K., & Jung, E. (2021). Anti-Inflammatory Activity and Mechanism of Isookanin, Isolated by Bioassay-Guided Fractionation from Bidens pilosa L. *Molecules*, *26*(2), 255. https://doi.org/10.3390/molecules26020255

Yan, Z., Chen, Z., Zhang, L., Wang, X., Zhang, Y., & Tian, Z. (2022). Bioactive polyacetylenes from Bidens pilosa L and their anti-inflammatory activity. *Natural Product Research*, *36*(24), 6353–6358. https://doi.org/10.1080/14786419.2022.2029432;WGROUP:STRING:PUBLICATION

Yang, H. L., Chen, S. C., Chang, N. W., Chang, J. M., Lee, M. L., Tsai, P. C., Fu, H. H., Kao, W. W., Chiang, H. C., Wang, H. H., & Hseu, Y. C. (2006). Protection from oxidative damage using Bidens pilosa extracts in normal human erythrocytes. *Food and Chemical Toxicology*, *44*(9), 1513–1521. https://doi.org/10.1016/J.FCT.2006.04.006

Yang, Y., Yu, K., & Zhang, Y. M. (2018). The Cardioprotective Effects of 4-O-(2″-O-acetyl-6″-O- P-coumaroyl-β-D-glucopyranosyl)-P-coumaric Acid (4-ACGC) on Chronic Heart Failure. *Iranian Journal of Pharmaceutical Research : IJPR*, *17*(2), 593.

Yi, J., Wu, J. G., Wu, Y. Bin, & Peng, W. (2016). Antioxidant and Anti-proliferative Activities of Flavonoids from Bidens pilosa L var radiata Sch Bip. *Tropical Journal of Pharmaceutical Research*, *15*(2), 341–348. https://doi.org/10.4314/TJPR.V15I2.17

Yuan, L. P., Chen, F. H., Ling, L., Dou, P. F., Bo, H., Zhong, M. M., & Xia, L. J. (2008). Protective effects of total flavonoids of Bidens pilosa L. (TFB) on animal liver injury and liver fibrosis. *Journal of Ethnopharmacology*, *116*(3), 539–546. https://doi.org/10.1016/J.JEP.2008.01.010

Yuniastri, R., Huzaimah, N., Estiasih, T., Martati, E., Tarmadi, D., Fatriasari, W., Arung, E. T., & Ismayati, M. (2022). *A COMPARATIVE EVALUATION OF THE ANTIOXIDANT ACTIVITY OF LOCAL PLANTS ORIGINATED FROM SUMENEP REGENCY, EAST JAVA, INDONESIA*. 87–94. https://doi.org/10.31788/RJC.2022.1558120
