# Supplementary material for: Exploring the phytochemical profile, antioxidant and anti-inflammatory potential of Bidens pilosa: A Systematic Review
Source: Front Pharmacol. 2025 Aug 1;16:1569527. doi: 10.3389/fphar.2025.1569527 (PMC12355053; doi:10.3389/fphar.2025.1569527)
Supplement: Supplementary file 2 [file Table2.docx]

**Supplementary Table 2:** **Phytocompounds identified in *Bidens pilosa* and their chemical formulae, molecular weights and chemical structures**

| S/N | Name | Mol. Formula | Mol.Wgt. (g/mol) | PubChem CID | Chemical Structure (2D) | Reference |
| --- | --- | --- | --- | --- | --- | --- |
| 1 | Palmitic acid | C16H32O2 | 256.42 | 985 | 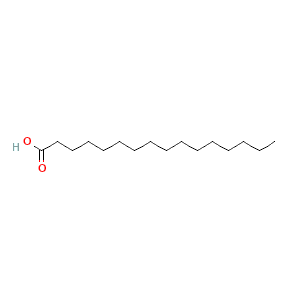 | (Quaglio et al., 2020) |
| 2 | Oleic acid | C18H34O2 | 282.5 | 445639 | 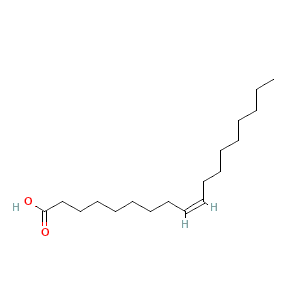 | (Quaglio et al., 2020) |
| 3 | Linoleic acid | C18H32O2 | 280.4 | 5280450 | 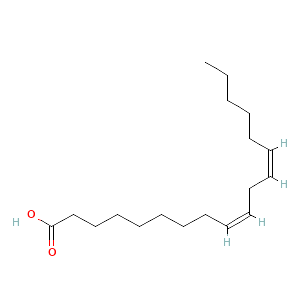 | (Quaglio et al., 2020) |
| 4 | Phytol | C20H40O | 296.5 | 5280435 | 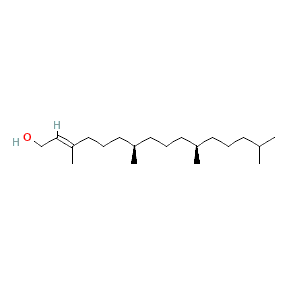 | (Quaglio et al., 2020) |
| 5 | Isookanin | C15H12O6 | 288.25 | 91196552 | 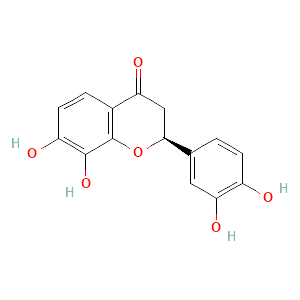 | (Fotso et al., 2014) |
| 6 | Ichthyothereol acetate | C16H16O3 | 256.3 | 73759958 | 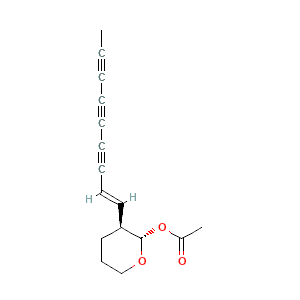 | (Yan et al., 2022) |
| 7 | Caffeic acid | C9H8O4 | 180.16 | 689043 | 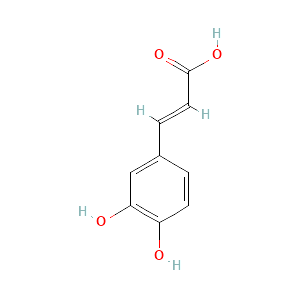 | (Horiuchi & Seyama, 2008) |
| 8 | 1-Caffeoylquinic acid | C16H18O9 | 354.31 | 10155076 | 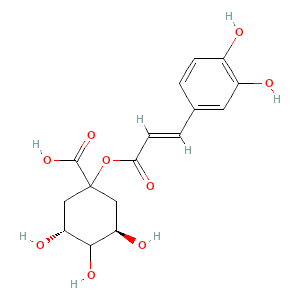 | (Horiuchi & Seyama, 2008) |
| 9 | Hyperin | C21H20O12 | 464.4 | 5281643 | 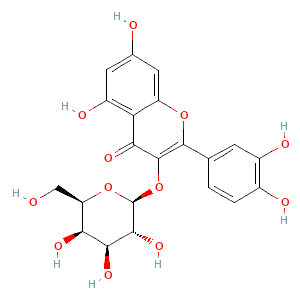 | (Horiuchi & Seyama, 2008) |
| 10 | Isoquercetin | C21H20O12 | 464.4 | 5280804 | 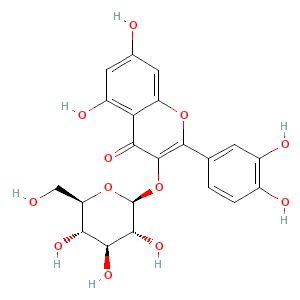 | (Horiuchi & Seyama, 2008) |
| 11 | 2-Undecenal | C11H20O | 168.28 | 5283356 | 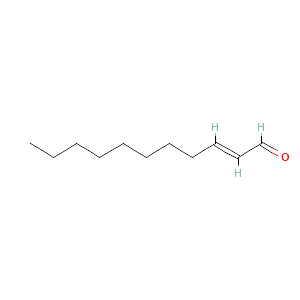 | (Abiodun et al., 2020) |
| 12 | O-Methylisourea | C2H6N2O | 74.08 | 75544 | 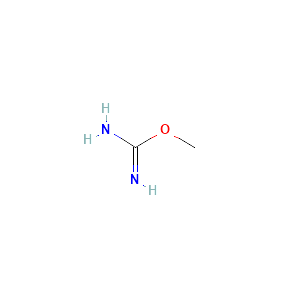 | (Abiodun et al., 2020) |
| 13 | Hexadecanoic acid methyl ester | C17H34O2 | 270.5 | 8181 | 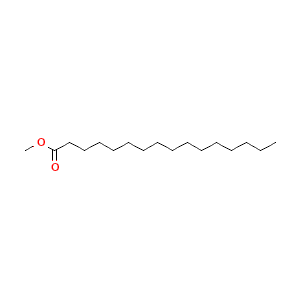 | (Abiodun et al., 2020) |
| 14 | Alpha-Pinene | C10H16 | 136.23 | 6654 | 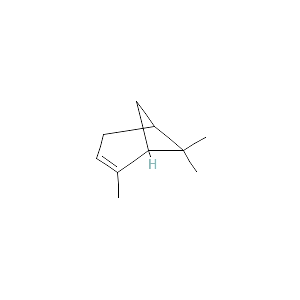 | (Goudoum et al., 2016) |
| 15 | Beta-Ocimene | C10H16 | 136.23 | 18756 | 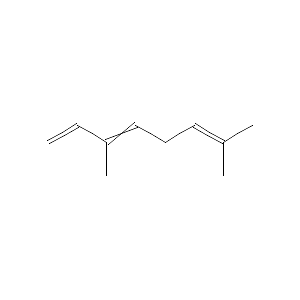 | (Goudoum et al., 2016) |
| 16 | Cadinene | C15H26 | 206.37 | 3032853 | 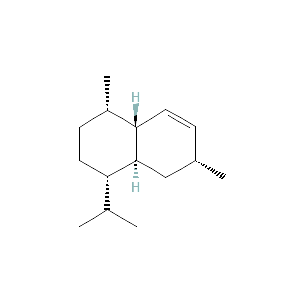 | (Goudoum et al., 2016) |
| 17 | Rutin | C27H30O16 | 610.5 | 5280805 | 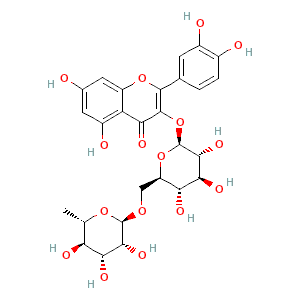 | (Nguyen et al., 2023) |
| 18 | Quercetin | C15H10O7 | 302.23 | 5280343 | 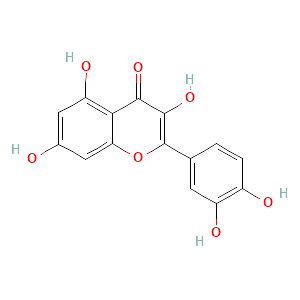 | (Fotso et al., 2014; Nguyen et al., 2023) |
| 19 | Beta-Caryophyllene | C15H24 | 204.35 | 5281515 | 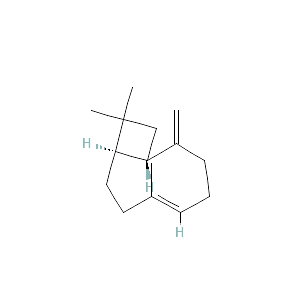 | (Deba et al., 2007; Goudoum et al., 2016) |
| 20 | Delta-Cadinene | C15H24 | 204.35 | 441005 | 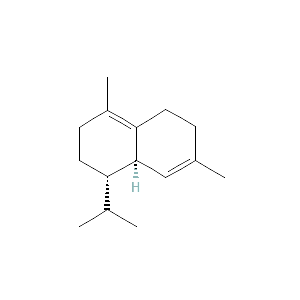 | (Deba et al., 2007) |
| 21 | Alpha-Pinene | C10H16 | 136.23 | 6654 | 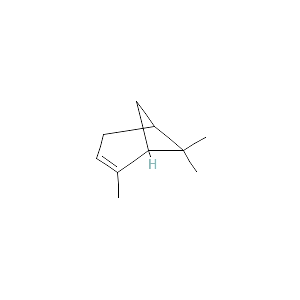 | (Deba et al., 2007) |
| 22 | Limonene | C10H16 | 136.23 | 22311 | 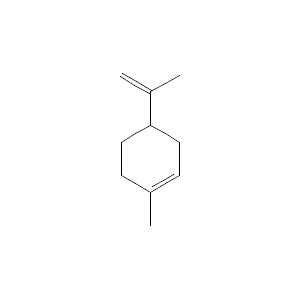 | (Deba et al., 2007) |
| 23 | Quercetin 3-O-rabinobioside | C27H30O16 | 610.5 | 10371536 | 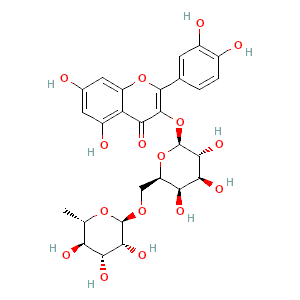 | (Chiang et al., 2004) |
| 24 | 5Z-Caffeoylquinic acid | C16H18O9 | 354.31 | 5315832 | 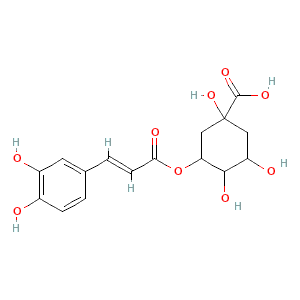 | (Chiang et al., 2004) |
| 25 | Quercetin 3,3′-dimethyl ether 7-O-β-D-glycopyranoside | C27H30O17 | 626.5 | 10121947 | 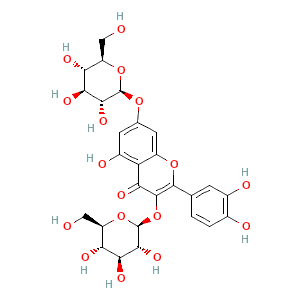 | (Kviecinski et al., 2011) |
| 26 | Catechin | C15H14O6 | 290.27 | 73160 | 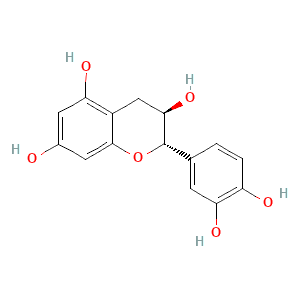 | (Singh et al., 2017) |
| 27 | Kaempferol | C15H10O6 | 286.24 | 5280863 | 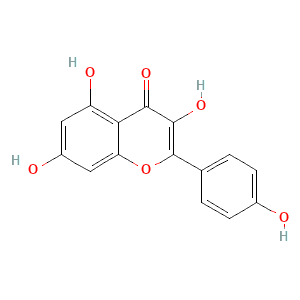 | (Singh et al., 2017) |
| 28 | Ferulic acid | C10H10O4 | 194.18 | 445858 | 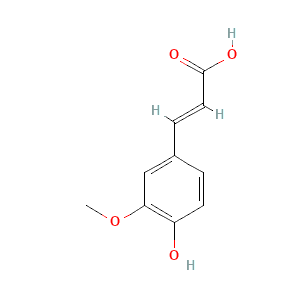 | (Singh et al., 2017) |
| 29 | Gallic acid | C7H6O5 | 170.12 | 370 | 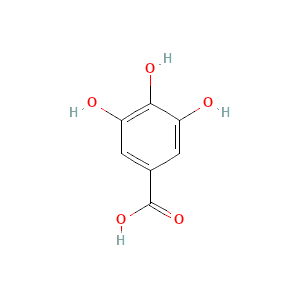 | (Singh et al., 2017) |
| 30 | Paclitaxel | C47H51NO14 | 853.9 | 36314 | 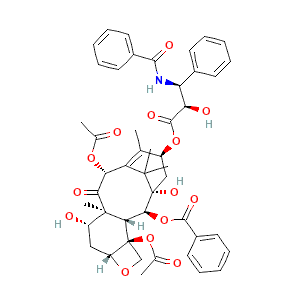 | (Singh et al., 2017) |
| 31 | Tannic acid | C76H52O46 | 1701.2 | 16129778 | 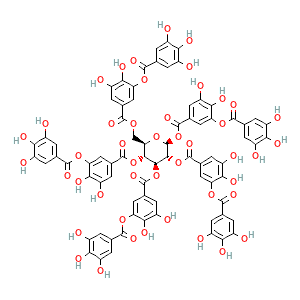 | (Mashinini et al., 2023) |
| 32 | Vanillin | C8H8O3 | 152.15 | 1183 | 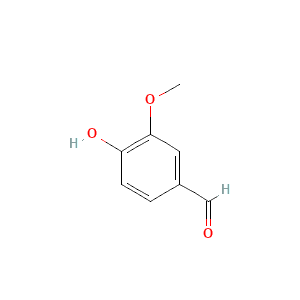 | (Mashinini et al., 2023) |
| 33 | Stigmasterol | C29H48O | 412.7 | 5280794 | 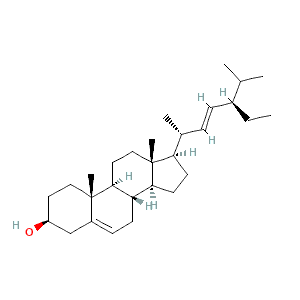 | (Rodríguez Mesa et al., 2023) |
| 34 | Beta-Sitosterol | C29H50O | 414.7 | 222284 | 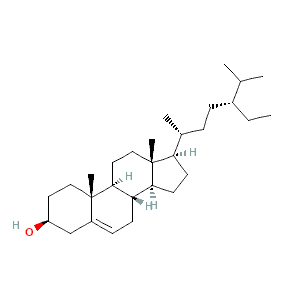 | (Rodríguez Mesa et al., 2023) |
| 35 | Ethyl Linoleate | C20H36O2 | 308.5 | 5282184 | 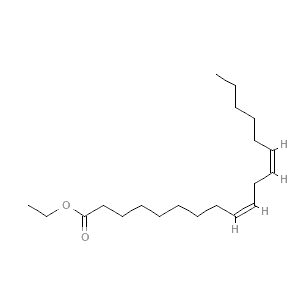 | (Rodríguez Mesa et al., 2023) |

Molecular formulae, molecular weight in grams/mole (**g/mol.**), PubChem Compound Identifiers (**CID**), and Two Dimensional (**2D**) Chemical Structures were presented as retrieved from the PubChem database: <https://pubchem.ncbi.nlm.nih.gov/> (Kim & Bolton, 2023)**.**

**References**

Abiodun, O. O., Sosanya, A. S., Nwadike, N., & Oshinloye, A. O. (2020). Beneficial effect of Bidens pilosa L. (Asteraceae) in a rat model of colitis. *Journal of Basic and Clinical Physiology and Pharmacology*, *31*(6), 1–9. https://doi.org/10.1515/jbcpp-2019-0166

Chiang, Y. M., Chuang, D. Y., Wang, S. Y., Kuo, Y. H., Tsai, P. W., & Shyur, L. F. (2004). Metabolite profiling and chemopreventive bioactivity of plant extracts from Bidens pilosa. *Journal of Ethnopharmacology*, *95*(2–3), 409–419. https://doi.org/10.1016/J.JEP.2004.08.010

Deba, F., Xuan, T. D., Yasuda, M., & Tawata, S. (2007). Chemical composition and antioxidant, antibacterial and antifungal activities of the essential oils from Bidens pilosa Linn. var. Radiata. *Food Control*, *19*(4), 346–352. https://doi.org/10.1016/J.FOODCONT.2007.04.011

Fotso, A. F., Longo, F., Djomeni, P. D. D., Kouam, S. F., Spiteller, M., Dongmo, A. B., & Savineau, J. P. (2014). Analgesic and antiinflammatory activities of the ethyl acetate fraction of Bidens pilosa (Asteraceae). *Inflammopharmacology*, *22*(2), 105–114. https://doi.org/10.1007/s10787-013-0196-2

Goudoum, A., Abdou, A. B., Ngamo, L. S. T., Ngassoum, M. B., & Mbofung, C. M. F. (2016). Antioxidant activities of essential oil of Bidens pilosa (Linn. Var. Radita) used for the preservation of food qualities in North Cameroon. *Food Science and Nutrition*, *4*(5), 671–678. https://doi.org/10.1002/FSN3.330

Horiuchi, M., & Seyama, Y. (2008). Improvement of the antiinflammatory and antiallergic activity of Bidens pilosa L. var. radiata SCHERFF treated with enzyme (Cellulosine). *Journal of Health Science*, *54*(3), 294–301. https://doi.org/10.1248/jhs.54.294

Kim, S., & Bolton, E. E. (2023). PubChem: A Large-Scale Public Chemical Database for Drug Discovery. *Open Access Databases and Datasets for Drug Discovery*, 39–66. https://doi.org/10.1002/9783527830497.CH2

Kviecinski, M. R., Felipe, K. B., Correia, J. F. G., Ferreira, E. A., Rossi, M. H., Gatti, F. de M., Filho, D. W., & Pedrosa, R. C. (2011). Brazilian Bidens pilosa linné yields fraction containing quercetin-derived flavonoid with free radical scavenger activity and hepatoprotective effects. *Libyan Journal of Medicine*, *6*(1), 1–8. https://doi.org/10.3402/LJM.V6I0.5651

Mashinini, P. P., Chihomvu, P., Pillay, M., & Takaidza, S. (2023). Phytochemical analysis and anti-mycobacterium activity of Bidens pilosa crude extracts. *Journal of Biotech Research*, *15*(September), 116–137.

Nguyen, T. H. D., Vu, D. C., Hanh, P. Q. P., Vo, X. T., Nguyen, V. C., Nguyen, T. N., Nguyen, L. L. P., & Baranyai, L. (2023). Comparative analysis of phenolic content and in vitro bioactivities of Bidens pilosa L. flowers and leaves as affected by extraction solvents. *Journal of Agriculture and Food Research*, *14*, 100879. https://doi.org/10.1016/J.JAFR.2023.100879

Quaglio, A. E. V., Cruz, V. M., Almeida-Junior, L. D., Costa, C. A. R. A., & Di Stasi, L. C. (2020). Bidens pilosa (Black Jack) Standardized Extract Ameliorates Acute TNBS-induced Intestinal Inflammation in Rats. *Planta Medica*, *86*(05), 319–330. https://doi.org/10.1055/A-1089-8342

Rodríguez Mesa, X. M., Contreras Bolaños, L. A., Modesti Costa, G., Mejia, A. L., & Santander González, S. P. (2023). A Bidens pilosa L. Non-Polar Extract Modulates the Polarization of Human Macrophages and Dendritic Cells into an Anti-Inflammatory Phenotype. *Molecules 2023, Vol. 28, Page 7094*, *28*(20), 7094. https://doi.org/10.3390/MOLECULES28207094

Singh, G., Passsari, A. K., Singh, P., Leo, V. V., Subbarayan, S., Kumar, B., Singh, B. P., & Kumar, N. S. (2017). *Pharmacological potential of Bidens pilosa L . and determination of bioactive compounds using UHPLC-QqQ LIT -MS / MS and GC / MS*. 1–16. https://doi.org/10.1186/s12906-017-2000-0

Yan, Z., Chen, Z., Zhang, L., Wang, X., Zhang, Y., & Tian, Z. (2022). Bioactive polyacetylenes from Bidens pilosa L and their anti-inflammatory activity. *Natural Product Research*, *36*(24), 6353–6358. https://doi.org/10.1080/14786419.2022.2029432;WGROUP:STRING:PUBLICATION
