# Supplementary material for: Exploring the phytochemical profile, antioxidant and anti-inflammatory potential of Bidens pilosa: A Systematic Review
Source: Front Pharmacol. 2025 Aug 1;16:1569527. doi: 10.3389/fphar.2025.1569527 (PMC12355053; doi:10.3389/fphar.2025.1569527)
Supplement: Supplementary file 3 [file Table3.docx]

**Supplementary Table 3: Antioxidant potentials of *B. pilosa* as reported in the included studies**

| **S/N** | **Extract/phytocompound** | **Mechanism of action** | **Outcome** | **Reference** |
| --- | --- | --- | --- | --- |
| 1 | Mucoadhesive formulation of *Bidens pilosa* (BP) and *Curcuma longa* (CL) extracts | Reduced malondialdehyde (MDA) levels | Restored oxidative stress parameters to levels comparable with the control group in 5-fluorouracil-induced mucositis model | (Bastos et al., 2016) |
| 2 | Ethyl acetate leave extract | NA | NA | (Fotso et al., 2014) |
| 3 | Lyophilized whole plant | NA | NA | (Chih et al., 1995) |
| 4 | Whole plant (Petroleum ether, dichloromethane, ethyl acetate and n-butanol fractions) | NA | NA | (Yan et al., 2022) |
| 5 | Aerial part, ethyl acetate and n-butanol fractions to isolate Isookanin | NA | NA | (Xin et al., 2021) |
| 6 | Dried leaves, methanolic extract and its isolated polyacetylene 2-O-b-D-glucosyltrideca-11E-en-3,5,7,9-tetrayn-1,2-diol (PA-1) | NA | NA | (Pereira et al., 1999) |
| 7 | Miyako Bidens pilosa extract | NA | NA | (Tsuruta et al., 2023) |
| 8 | Musashino Miyako Bidens pilosa (MMBP) extract | NA | NA | (Horiuchi & Seyama, 2008) |
| 9 | Extract obtained by nonpolar carbon dioxide super-critical extraction | NA | NA | (Quaglio et al., 2020) |
| 10 | FITOPROT formulation containing curcuminoids and *B. pilosa* extract | NA | NA | (Arantes et al., 2021) |
| 11 | Butanol fraction | NA | NA | (Chang et al., 2005) |
| 12 | Ethanol, n-butanol, ethyl acetate, and petroleum ether fractions. Finally utilized ethyl acetate fractions | NA | NA | (Y. Yang et al., 2018) |
| 13 | Aqueous extract | NA | NA | (Pegoraro et al., 2018) |
| 14 | Ecobidens® (B. pilosa glycolic extract) | Reduced malondialdehyde (MDA) levels | B. pilosa extract reduced MDA activities in 5-Fluorouracil (5-FU)-induced intestinal mucositis mice models, demonstrating protective effects compared to the 5-FU group. | (de Ávila et al., 2015) |
| 15 | FITOPROT A: A mucoadhesive formulation composed of 10 mg/mL curcuminoid extract combined with 20% v/v Bidens pilosa L. extract. FITOPROT B: A mucoadhesive formulation containing 20 mg/mL curcuminoid extract along with 40% v/v Bidens pilosa L. extract. | FITOPROT A and FITOPROT B did not show significant differences in mean salivary concentrations of MDA, indicating that both formulations had similar effects on oxidative stress levels in participants. | FITOPROT was both safe and well-tolerated at the tested doses, making it a viable candidate for further assessment in a phase II trial as a potential treatment for oral mucositis. | (Santos Filho et al., 2018) |
| 16 | Bidens pilosa Tea (PT) | *In-vitro:* PT extract exhibited the highest total phenolic content (TPC) and demonstrated superior antioxidant capacity, effectively scavenging DPPH and ABTS radicals, and showing the strongest reducing power among all tea extracts studied. *In-vivo:* *B. pilosa* tea (PT) treatment significantly restored antioxidant parameters in high oxalate diet (KOx) rats by mitigating reductions in glutathione (GSH) and antioxidant enzymes, and lowering elevated malondialdehyde (MDA) levels, demonstrating the strongest protective effect among the tea extracts. | *B. pilosa* tea reduced inflammation, glutathione redox equilibrium, prevent peroxidative damage, and replenish renal tissue antioxidants | (Mohamed et al., 2024) |
| 17 | Aqueous and methanolic extracts | *Bidens pilosa* methanolic and aqueous extracts were among the first five plants with the highest phenolic compounds content. | *Bidens pilosa* leaves extract exhibited strong antioxidant activity, effectively scavenging DPPH radicals and contained high levels of phenolic compounds, suggesting potential benefits for reducing oxidative stress | (Akula & Odhav, 2008) |
| 18 | NA | *B. pilosa* demonstrated high levels of carbohydrates, carotenoids, ascorbic acid, and phenolic compounds. It also showed increased activity of antioxidant enzymes such as superoxide dismutase (SOD), catalase (CAT), and peroxidase (POD) at this stage, indicating strong antioxidant potential. | *B. pilosa* can significantly contribute to nutritional security and health, particularly in rural communities | (Tesfay et al., 2016) |
| 19 | Ethanol and aqua dest Leave extract (B-1) | DPPH test results indicated that B-1 exhibited the strongest antioxidant properties, with an IC50 value of approximately 6.43%. These antioxidant effects were influenced by its high phenolic content | B-1 extract shows promise as an antioxidant additive for applications in the food, cosmetic, and advanced materials industries | (Yuniastri et al., 2022) |
| 20 | Methanolic extract | Reduced colonic MDA levels and increased glutathione levels in colitic rats by 3–4 fold | *B. pilosa* can reduce colonic lipid peroxidation and oxidative stress indicating protective effects against colitis in rats | (Abiodun et al., 2020) |
| 21 | Methanol 50%/formic acid 1% extract | Bidens pilosa exhibited strong antioxidant activity across DPPH, FRAP, and ABTS+ assays, demonstrating effective radical scavenging and reducing power alongside other plants like *S. cordatum* | B. pilosa expressed strong antioxidant activity especially in DPPH and ABTS systems. | (Nxumalo et al., 2023) |
| 22 | Chloroform, ethyl acetate, formic acid/ethyl acetate, methanol, and water | *B. pilosa* showed strong antioxidant activity in DPPH assay by scavenging free radicals, thereby neutralizing them and preventing oxidative damage to cells. | *B.pilosa* expressed antioxidant activity due to moderate flavonoids and terpenoids content | (Shandukani et al., 2018) |
| 23 | Leave essential oil | *B. pilosa* exhibited strong antioxidant activity in DPPH, reducing power, and β-carotene-linoleate assays by scavenging free radicals and reducing oxidative stress through its various phytochemical constituents. | *B. pilosa* demonstrated significant antioxidant and free radical-scavenging activity, making it beneficial for food preservation and traditional medicine despite weaker effects compared to BHT. | (Goudoum et al., 2016) |
| 24 | Mutarase K isolated from Acetonic fraction | NA | NA | (Mota et al., 2019) |
| 25 | Crude, ethanaol and methanol extracts | *Bidens pilosa* demonstrated strong antioxidant activity, exhibiting high % DPPH inhibition and significant FRAP values, indicating its effective capacity to scavenge free radicals and reduce oxidative stress. | B. pilosa showed high antioxidant activity owing to a rich phytochemical profile; including alkaloids, phenols, flavonoids, phyto-steroids, glycosides, and saponins, with the highest total phenolic content (TPC) and total flavonoid content (TFC) among the samples. | (Phiri et al., 2024) |
| 26 | Water, mehanol, aceton and ethyl acetate extracts | Flower and leaf extracts exhibited significant antioxidant activity by effectively scavenging free radicals, as evidenced by their ability to inhibit ABTS and DPPH radicals. | The methanolic and acetonic extracts showed the strongest antioxidant effects, with inhibition percentages exceeding 70%. | (Nguyen et al., 2023) |
| 27 | 75% ethanol for sterilization; Liquid nitogen + dimethyl sulfoxide (DMSO) for dissolving the ground powder | NA | NA | (Said et al., 2024) |
| 28 | Methanol extract | *B. pilosa* exhibited antioxidant properties by scavenging of free radicals in DPPH, ABTS and FRAP systems | The methanol extracts of *B. pilosa*, particularly from the leaves, exhibit significant radical scavenging and reducing activities, highlighting their potential as natural antioxidants. | (Angelini et al., 2021) |
| 29 | Aqueous extract | *B. pilosa* showed antioxidant activity by scavenging radicals in DPPH, ABTS and Nitic oxide systems and reduced the oxidation of LDL | Among the 11 Thai weeds, *B. pilosa* exhibited the strongest radical scavenging activity except in ABTS system. This effect was attributed to its high phenolic content | (U-Yatung et al., 2020) |
| 30 | Aqueous extract | *B. pilosa* showed highest radical scavenging activity in DPPH and FRAP systems | Among the 6 vegetables studied, *B. pilosa* had the highest flavonoid content and highest total antioxidant properties. | (Oduntan et al., 2018) |
| 31 | Ethanol-water extract | B. pilosa exhibited its antiradical activity by donating electrons to neutralize DPPH and ABTS radicals, thereby reducing oxidative stress. It reduced MDA levels | B. pilosa leaf demonstrated higher antiradical activity against DPPH and ABTS radicals compared to *M. oleifera* leaf extract and the standard butylated hydroxytoluene (BHT). It reduced lipid peroxidation in beef samples. | (Falowo et al., 2017) |
| 32 | Methanol extract | B. pilosa showed strong antioxidant activity by scavaging radicals in DPPH and ABTS systems | *B. pilosa* demonstrated effective scavenging of DPPH and ABTS radicals, and it showed high levels of phenolics and flavonoids, indicating its strong antioxidant potential. | (Singh et al., 2017) |
| 33 | Aqueous and ethanol extracts | *B. pilosa* had strong scavenging activity on radicals in ABTS and FRAP systems | The extracts of *Bidens pilosa* demonstrated enhanced antioxidant activity, with ethanol extracts showing higher concentrations of total polyphenols and flavonols compared to water extracts. This resulted in a more potent ability to scavenge free radicals and reduce oxidative stress. | (Idris et al., 2023) |
| 34 | NA. Powered plant was mixed in feed | *B. pilosa* significantly enhanced the expression levels of antioxidant enzymes (CAT and SOD1) and peptide transporter 1 (PepT1) in various treatment groups | *B. pilosa* contain bioactive compounds that upregulate gene expression and improve antioxidant defense mechanisms in *Eimeria* infected chickens | (Memon et al., 2020) |
| 35 | Ethanol extract | In-vitro*, B. pilosa* exhibited antioxidant capacity across four test methods DPPH, ABTS, FRAP and Total antioxidant. It demonstrated significant free radical scavenging ability and reducing activity, particularly in the ABTS assay. *In-vivo*, it increased the lifespan of fruit flies under oxidative stress, extending their life by 1.41 times with Paraquat and 1.25 times with H2O2 compared to controls. | The study found that B. pilosa L. extract demonstrated strong antioxidant capacity and prolonged fruit fly lifespan under oxidative stress, supporting its potential use in functional foods and therapies | (Son et al., 2022) |
| 36 | crude, acetone, methanolic, hexane and ethanolic extracts. | *B. pilosa* extracts from dried plant material showed stronger antioxidant activity than ascorbic acid, and better scavenging activity than fresh plant material in DPPH and ABTS | B. pilosa exerted antioxidant potential, significant for drug development, especially for mycobacterial infections | (Mashinini et al., 2023) |
| 37 | FITOPROT formulation containing curcuminoids and *B. pilosa* extract | NA | NA | (Arantes et al., 2021) |
| 38 | Diethyl ether extract | In the DPPH test, the essential oils from the leaves and flowers of *B. pilosa* exhibited superior antioxidant activity, outperforming all tested aqueous extracts. In the β-carotene bleaching test, both the extracts and essential oils effectively inhibited the oxidation of linoleic acid. | *B. pilosa* exhibits antioxidant activity, suggesting it could serve as a natural source of preservatives in the food industry and related sectors. | (Deba et al., 2007) |
| 39 | Crude, ethanol, ethyl acetate and n-buthanol fractions | *B. pilosa* extracts demonstrated significant radical scavenging activity, with the ethyl acetate (EA) and butanol (BuOH) fractions showing effective inhibition of DPPH radicals, although less potent than quercetin and ascorbic acid. In the superoxide scavenging test, the EA and BuOH fractions of *B. pilosa* exhibited notable scavenging activity, with quercetin being the most effective. | *B. pilosa* extracts, particularly the EA and BuOH fractions, possess strong antioxidant properties, effectively scavenging free radicals and superoxide, suggesting potential for use in antioxidant therapies. | (Chiang et al., 2004) |
| 40 | Ethanolic fraction | In all isolated compounds, quercetin had the highest antioxidant activity in DPPH and ABTS system | B. Pilosa is rich in flavonoid compounds with notable antioxidant properties, indicating its potential as a health supplement and a readily accessible source of natural antioxidants for colon cancer prevention. | (Yi et al., 2016) |
| 41 | Honey extract | Honey derived from B. pilosa exhibited notably higher levels of total phenolic and flavonoid compounds than other varieties. Additionally, it demonstrated superior scavenging activity against DPPH and hydroxyl radicals, as well as increased reducing power. | These results indicate that the antioxidant levels in honey are linked to its total phenolic and flavonoid content, highlighting B. pilosa as a promising source of antioxidant activity. | (Liu et al., 2013) |
| 42 | Aqueous methanolic extract | *B. pilosa* had the second-best performing extract in DPPH, Reducing power, β-carotene-linoleate system and phospholipid peroxidation systems. It expressed antioxidant activities and strong radical scavenging potential. It reduced phospholipid peroxidation in TBARS system | *Cleome gynandra* and *B. pilosa* expressed good antioxidant and lipid peroxidation reducing potentials. This was time-dependent, highlighting them as good botanical drug candidates. | (Muchuweti et al., 2007) |
| 43 | Chloroform, ethyl acetate and methanolic fractions | The ethyl acetate fraction (f-EtOAc) from *B. pilosa* demonstrated significant antioxidant activity by improving FRAP and GSH levels and modulating CAT activity. The ethyl acetate fraction (f-EtOAc) of *B. pilosa* demonstrated the highest antioxidant effect across all measured parameters in DPPH, Hydroxyl radical scavenging and lipid peroxidation, making it the most effective fraction in scavenging free radicals and inhibiting lipid peroxidation. | The ethyl acetate fraction (f-EtOAc) of Bidens pilosa effectively extracts key hepatoprotective and antioxidant constituents, supporting its traditional use in Brazil. Further evaluation of its therapeutic efficacy and safety for liver disease treatment is warranted. | (Kviecinski et al., 2011) |
| 44 | Musashino Miyako Bidens pilosa (MMBP) extract | Treatment with MMBP B. pilosa's formulation, rutin, or sucralfate, had potent antioxidative activity, inhibited the increases in the levels of thiobarbituric acid reactivesubstances (TBARS) in the gastric mucosal lesions | MMBP prevents the progression of acute gastric mucosal lesions, possibly by suppressing oxidative stress in the gastric mucosa. | (Horiuchi et al., 2010) |
| 45 | Aqueous metahnolic and methanolic extracts | *B. pilosa* exhibited significant antioxidant properties, characterized by a high total phenolic content (gallic acid equivalence), indicating strong antioxidant potential. Its methanolic extract effectively scavenged DPPH radicals, comparable to standard antioxidants like ascorbic acid and catechin. Additionally, it demonstrated notable reducing power, reflecting its ability to donate electrons and mitigate oxidative stress. The extract also effectively delayed β-carotene bleaching, showcasing its capacity to protect against lipid oxidation. | *B. pilosa* exhibited strong antioxidant activities, supporting its potential use as a natural source of antioxidants in combating oxidative stress. | (Chipurura et al., 2013) |
| 46 | Acetone, aqueous and methanol extracts | *B. pilosa* demonstrated significant antioxidant properties, exhibiting high Ferric Reducing Antioxidant Power (FRAP) and effective DPPH radical scavenging activity, comparable to standard antioxidants like ascorbic acid and BHT. Additionally, it achieved substantial ABTS radical scavenging inhibition, underscoring its potential as a powerful natural antioxidant. | *B. pilosa* exhibited strong antioxidant capabilities and a favorable nutritional profile, supporting its potential use as a natural source of health benefits. | (Adedapo, 2011) |
| 47 | Crude extract | Total flavonoids from *B. pilosa (TFB)* effectively mitigated CCl₄-induced liver damage in mice by decreasing hepatic MDA content, and restoring hepatic SOD and GSH-Px activities. In rats with liver fibrosis, these flavonoids significantly inhibited NF-κB activation and histopathological analysis indicated a reduction in liver injury severity | TFB exhibited protective and therapeutic effects against liver injury in animals, likely due to its antioxidant properties and the inhibition of NF-κB activation. | (Yuan et al., 2008) |
| 48 | Optimized ethanol extract | The optimized ethanol extract of *B. pilosa* demonstrated significant antioxidant activity, for DPPH and ABTS. There was a strong correlation between total flavonoid content (TFC), total phenolic content (TPC), and the antioxidant activity of the extracts. | This study highlighted the potential of B. pilosa as a natural source of antioxidants, which may serve as a healthier alternative to synthetic antioxidants, given the growing interest in natural substances for their health benefits | (Cortés-Rojas et al., 2011) |
| 49 | Petroleum ether, ethyl acetate, n-Butanol and water fractions | The ethyl acetate fraction (EE-BP) exhibited the highest antioxidant activity (through radical scavenging) among all fractions in the DPPH, FRAP, and ABTS assays | Bidens pilosa (BP) contains high levels of phenols and flavonoids, which contribute to its significant antioxidant activities. These properties, along with the induction of apoptosis in RKO cells, suggest that BP may play a critical role in the prevention and treatment of colorectal cancer, partly due to the active compound BP-6 (containing 5,7,40-trihydroxy-3,30-dimethyl-flavonol) | (Wu et al., 2012) |
| 50 | Ethanol and ethyl acetate extracts | Extracts of *B. pilosa* diminished oxidative hemolysis and lipid/protein peroxidation in erythrocytes in a concentration- and time-dependent manner. Additionally, the extracts helped maintain superoxide dismutase (SOD) activity and prevented the depletion of cytosolic glutathione (GSH) and ATP in erythrocytes. | *B. pilosa* may have cytoprotective and antioxidant properties in living systems | (H. L. Yang et al., 2006) |

**NA:** Not applicable; **DPPH**: 2,2-diphenyl-1-picrylhydrazyl; **ABTS**: 2,2'-azinobis(3-ethylbenzothiazoline-6-sulfonic acid); **FRAP**: Ferric Reducing Antioxidant Power; **TBARS**: Thiobarbituric Acid Reactive Substances; **SOD**: Superoxide Dismutase; **CAT**: Catalase; **MDA**: Malondialdehyde; **LPO**: Lipid Peroxidation; **MMT**: Methyl Methacrylate Trimer; **NF-κB**: Nuclear Factor kappa-light-chain-enhancer of activated B cells; **GSH**: Glutathione; **GSH-Px**: Glutathione Peroxidase; **TPC**: Total Phenolic Content; **TFC**: Total Flavonoid Content.

**References**

Abiodun, O. O., Sosanya, A. S., Nwadike, N., & Oshinloye, A. O. (2020). Beneficial effect of Bidens pilosa L. (Asteraceae) in a rat model of colitis. *Journal of Basic and Clinical Physiology and Pharmacology*, *31*(6), 1–9. https://doi.org/10.1515/jbcpp-2019-0166

Adeolu Adedapo, F. J. and A. A. (2011). *Comparison of the nutritive value and biological activities of the acetone, methanol and water extracts of the leaves of Bidens pilosa and Chenopodium album - PubMed*. Acta Poloniae Pharmaceutica ñ Drug Research, Vol. 68 No. 1 Pp. 83ñ92, 2011.

Akula, U. S., & Odhav, B. (2008). *In vitro 5-Lipoxygenase inhibition of polyphenolic antioxidants from undomesticated plants of South Africa*. *2*(9), 207–212.

Angelini, P., Matei, F., Flores, G. A., Pellegrino, R. M., Vuguziga, L., Venanzoni, R., Tirillini, B., Emiliani, C., Orlando, G., Menghini, L., & Ferrante, C. (2021). Metabolomic Profiling, Antioxidant and Antimicrobial Activity of Bidens pilosa. *Processes 2021, Vol. 9, Page 903*, *9*(6), 903. https://doi.org/10.3390/PR9060903

Arantes, D. A. C., da Silva, A. C. G., Freitas, N. M. A., Lima, E. M., de Oliveira, A. C., Marreto, R. N., Mendonça, E. F., & Valadares, M. C. (2021). Safety and efficacy of a mucoadhesive phytomedication containing curcuminoids and Bidens pilosa L. extract in the prevention and treatment of radiochemotherapy-induced oral mucositis: Triple-blind, randomized, placebo-controlled, clinical trial. *Head and Neck*, *43*(12), 3922–3934. https://doi.org/10.1002/HED.26892;PAGEGROUP:STRING:PUBLICATION

Arantes, D. A. C., da Silva, A. C. G., Lima, E. M., Alonso, E. C. P., Marreto, R. N., Mendonça, E. F., Valadares, M. C., & Batista, A. C. (2021). Biological effects of formulation containing curcuminoids and *Bidens Pilosa L*. in oral carcinoma cell line. *Brazilian Oral Research*, *35*, e063. https://doi.org/10.1590/1807-3107BOR-2021.VOL35.0063

Bastos, C. C. C., Ávila, P. H. M. de, Filho, E. X. dos S., Ávila, R. I. de, Batista, A. C., Fonseca, S. G., Lima, E. M., Marreto, R. N., Mendonça, E. F. de, & Valadares, M. C. (2016). Use of Bidens pilosa L. (Asteraceae) and Curcuma longa L. (Zingiberaceae) to treat intestinal mucositis in mice: Toxico-pharmacological evaluations. *Toxicology Reports*, *3*, 279–287. https://doi.org/10.1016/j.toxrep.2015.10.013

Chang, C. L. T., Kuo, H. K., Chang, S. L., Chiang, Y. M., Lee, T. H., Wu, W. M., Shyur, L. F., & Yang, W. C. (2005). The distinct effects of a butanol fraction of Bidens pilosa plant extract on the development of Th1-mediated diabetes and Th2-mediated airway inflammation in mice. *Journal of Biomedical Science*, *12*(1), 79–89. https://doi.org/10.1007/S11373-004-8172-X/METRICS

Chiang, Y. M., Chuang, D. Y., Wang, S. Y., Kuo, Y. H., Tsai, P. W., & Shyur, L. F. (2004). Metabolite profiling and chemopreventive bioactivity of plant extracts from Bidens pilosa. *Journal of Ethnopharmacology*, *95*(2–3), 409–419. https://doi.org/10.1016/J.JEP.2004.08.010

Chih, H. W., Lin, C. C., & Tang, K. S. (1995). Anti-inflammatory activity of Taiwan folk medicine “ham-hong-chho” in rats. *The American Journal of Chinese Medicine*, *23*(3–4), 273–278. https://doi.org/10.1142/S0192415X95000328;ISSUE:ISSUE:10.1142/AJCM.23.ISSUE-03N04;PAGEGROUP:STRING:PUBLICATION

Chipurura, B., Muchuweti, M., & Bhebhe, M. (2013). An assessment of the phenolic content, composition and antioxidant capacity of selected indigenous vegetables of Zimbabwe. *Acta Horticulturae*, *979*, 611–620. https://doi.org/10.17660/ACTAHORTIC.2013.979.66

Cortés-Rojas, D. F., Souza, C. R. F., & Oliveira, W. P. (2011). Optimisation of the extraction of phenolic compounds and antioxidant activity from aerial parts of Bidens pilosa L. using response surface methodology. *International Journal of Food Science and Technology*, *46*(11), 2420–2427. https://doi.org/10.1111/J.1365-2621.2011.02765.X

de Ávila, P. H. M., de Ávila, R. I., dos Santos Filho, E. X., Cunha Bastos, C. C., Batista, A. C., Mendonça, E. F., Serpa, R. C., Marreto, R. N., da Cruz, A. F., Lima, E. M., & Valadares, M. C. (2015). Mucoadhesive formulation of Bidens pilosa L. (Asteraceae) reduces intestinal injury from 5-fluorouracil-induced mucositis in mice. *Toxicology Reports*, *2*, 563. https://doi.org/10.1016/J.TOXREP.2015.03.003

Deba, F., Xuan, T. D., Yasuda, M., & Tawata, S. (2007). Chemical composition and antioxidant, antibacterial and antifungal activities of the essential oils from Bidens pilosa Linn. var. Radiata. *Food Control*, *19*(4), 346–352. https://doi.org/10.1016/J.FOODCONT.2007.04.011

Falowo, A. B., Muchenje, V., Hugo, A., Aiyegoro, O. A., & Fayemi, P. O. (2017). Antioxidant activities of Moringa oleifera L. and Bidens pilosa L. leaf extracts and their effects on oxidative stability of ground raw beef during refrigeration storage. *CyTA - Journal of Food*, *15*(2), 249–256. https://doi.org/10.1080/19476337.2016.1243587

Fotso, A. F., Longo, F., Djomeni, P. D. D., Kouam, S. F., Spiteller, M., Dongmo, A. B., & Savineau, J. P. (2014). Analgesic and antiinflammatory activities of the ethyl acetate fraction of Bidens pilosa (Asteraceae). *Inflammopharmacology*, *22*(2), 105–114. https://doi.org/10.1007/s10787-013-0196-2

Goudoum, A., Abdou, A. B., Ngamo, L. S. T., Ngassoum, M. B., & Mbofung, C. M. F. (2016). Antioxidant activities of essential oil of Bidens pilosa (Linn. Var. Radita) used for the preservation of food qualities in North Cameroon. *Food Science and Nutrition*, *4*(5), 671–678. https://doi.org/10.1002/FSN3.330

Horiuchi, M., & Seyama, Y. (2008). Improvement of the antiinflammatory and antiallergic activity of Bidens pilosa L. var. radiata SCHERFF treated with enzyme (Cellulosine). *Journal of Health Science*, *54*(3), 294–301. https://doi.org/10.1248/jhs.54.294

Horiuchi, M., Wachi, H., & Seyama, Y. (2010). Effects of Bidens pilosa L. var. radiata Scherff on experimental gastric lesion. *Journal of Natural Medicines*, *64*(4), 430–435. https://doi.org/10.1007/s11418-010-0426-5

Idris, O. A., Kerebba, N., Horn, S., Maboeta, M. S., & Pieters, R. (2023). Phytochemical-Based Evidence of the Health Benefits of Bidens Pilosa Extracts and Cytotoxicity. *Chemistry Africa*, *6*(4), 1767–1788. https://doi.org/10.1007/s42250-023-00626-2

Kviecinski, M. R., Felipe, K. B., Correia, J. F. G., Ferreira, E. A., Rossi, M. H., Gatti, F. de M., Filho, D. W., & Pedrosa, R. C. (2011). Brazilian Bidens pilosa linné yields fraction containing quercetin-derived flavonoid with free radical scavenger activity and hepatoprotective effects. *Libyan Journal of Medicine*, *6*(1), 1–8. https://doi.org/10.3402/LJM.V6I0.5651

Liu, J. R., Ye, Y. L., Lin, T. Y., Wang, Y. W., & Peng, C. C. (2013). Effect of floral sources on the antioxidant, antimicrobial, and anti-inflammatory activities of honeys in Taiwan. *Food Chemistry*, *139*(1–4), 938–943. https://doi.org/10.1016/j.foodchem.2013.02.015

Mashinini, P. P., Chihomvu, P., Pillay, M., & Takaidza, S. (2023). Phytochemical analysis and anti-mycobacterium activity of Bidens pilosa crude extracts. *Journal of Biotech Research*, *15*(September), 116–137.

Memon, F. Q., Yang, Y., Lv, F., Soliman, A. M., Chen, Y., Sun, J., Wang, Y., Zhang, G., Li, Z., Xu, B., Gadahi, J. A., & Si, H. (2020). *Effects of probiotics and Bidens pilosa on the performance and gut health of chicken during induced E. tenella infection*. https://doi.org/10.21203/RS.3.RS-18318/V1

Mohamed, R. S., Ramadan, M. M., Fouda, K., Ghanem, K. Z., Omara, E. A., & Abdel-Aziz, S. A. (2024). Preventive Impacts of Black Tea, Green Tea and Bidens pilosa on Renal Stone Formation in Rats: Antioxidant and Anti-inflammatory Pathways. *Egyptian Journal of Chemistry*, *67*(12), 423–432. https://doi.org/10.21608/EJCHEM.2024.317701.10337

Mota, C. M., Santiago, F. M., Cardoso, M. de R. D., Rostkowska, C., de Oliveira, T. C., Silva, D. A. de O., Pirovani, C. P., Mineo, T. W. P., & Mineo, J. R. (2019). Acetonic fraction of Bidens pilosa enriched for maturase K is able to control cerebral parasite burden in mice experimentally infected with Toxoplasma gondii. *Frontiers in Veterinary Science*, *6*(MAR), 1–13. https://doi.org/10.3389/fvets.2019.00055

Muchuweti, M., Mupure, C., Ndhlala, A., Murenje, T., & Benhura, M. A. N. (2007). Screening of antioxidant and radical scavenging activity of Vigna ungiculata, Bidens pilosa and Cleome gynandra. *American Journal of Food Technology*, *2*(3), 161–168. https://doi.org/10.3923/AJFT.2007.161.168

Nguyen, T. H. D., Vu, D. C., Hanh, P. Q. P., Vo, X. T., Nguyen, V. C., Nguyen, T. N., Nguyen, L. L. P., & Baranyai, L. (2023). Comparative analysis of phenolic content and in vitro bioactivities of Bidens pilosa L. flowers and leaves as affected by extraction solvents. *Journal of Agriculture and Food Research*, *14*, 100879. https://doi.org/10.1016/J.JAFR.2023.100879

Nxumalo, K. A., Aremu, A. O., & Fawole, O. A. (2023). Metabolite profiling, antioxidant and antibacterial properties of four medicinal plants from Eswatini and their relevance in food preservation. *South African Journal of Botany*, *162*, 719–729. https://doi.org/10.1016/J.SAJB.2023.10.008

Oduntan, A. O., Fasoyiro, S. B., Akinfasoye, J. A., Adeboyejo, F. O., & Akintoye, H. A. (2018). Antioxidant and proximate properties of underutilized vegetables in western Nigeria. *Acta Horticulturae*, *1225*, 255–260. https://doi.org/10.17660/ACTAHORTIC.2018.1225.35

Pegoraro, C. M. R., Nai, G. A., Garcia, L. A., Serra, F. de M., Alves, J. A., Chagas, P. H. N., Oliveira, D. G. de, & Zocoler, M. A. (2018). Protective effects of Bidens pilosa on hepatoxicity and nephrotoxicity induced by carbon tetrachloride in rats. *Drug and Chemical Toxicology*, *44*(1), 64–74. https://doi.org/10.1080/01480545.2018.1526182;WGROUP:STRING:PUBLICATION

Pereira, R. L. C., Ibrahim, T., Lucchetti, L., Da Silva, A. J. R., & De Moraes, V. L. G. (1999). Immunosuppressive and anti-inflammatory effects of methanolic extract and the polyacetylene isolated from Bidens pilosa L. *Immunopharmacology*, *43*(1), 31–37. https://doi.org/10.1016/S0162-3109(99)00039-9

Phiri, H., Lumai, A., Zombe, K., & Nyirenda, J. (2024). Evaluation of antioxidant activity of selected wild fruits and vegetables from Zambia. *Food and Humanity*, *3*, 100390. https://doi.org/10.1016/J.FOOHUM.2024.100390

Quaglio, A. E. V., Cruz, V. M., Almeida-Junior, L. D., Costa, C. A. R. A., & Di Stasi, L. C. (2020). Bidens pilosa (Black Jack) Standardized Extract Ameliorates Acute TNBS-induced Intestinal Inflammation in Rats. *Planta Medica*, *86*(05), 319–330. https://doi.org/10.1055/A-1089-8342

Said, W., Khattab, A. A., Hamed, S. A., Abo-Elmaaty, S. A., & Khalil, H. (2024). Identification of Bioactive and Anticancer Properties of Bidens Pilosa in-vitro Evidence. *Asian Pacific Journal of Cancer Prevention : APJCP*, *25*(10), 3551. https://doi.org/10.31557/APJCP.2024.25.10.3551

Santos Filho, E. X. dos, Arantes, D. A. C., Oton Leite, A. F., Batista, A. C., Mendonça, E. F. de, Marreto, R. N., Naves, L. N., Lima, E. M., & Valadares, M. C. (2018). Randomized clinical trial of a mucoadhesive formulation containing curcuminoids (Zingiberaceae) and Bidens pilosa Linn (Asteraceae) extract (FITOPROT) for prevention and treatment of oral mucositis - phase I study. *Chemico-Biological Interactions*, *291*, 228–236. https://doi.org/10.1016/J.CBI.2018.06.010

Shandukani, P. D., Tshidino, S. C., Masoko, P., & Moganedi, K. M. (2018). Antibacterial activity and in situ efficacy of Bidens pilosa Linn and Dichrostachys cinerea Wight et Arn extracts against common diarrhoea-causing waterborne bacteria. *BMC Complementary and Alternative Medicine*, *18*(1). https://doi.org/10.1186/S12906-018-2230-9

Singh, G., Passsari, A. K., Singh, P., Leo, V. V., Subbarayan, S., Kumar, B., Singh, B. P., lalhlenmawia, H., & Kumar, N. S. (2017). Pharmacological potential of Bidens pilosa L. and determination of bioactive compounds using UHPLC-QqQLIT-MS/MS and GC/MS. *BMC Complementary and Alternative Medicine*, *17*(1), 1–16. https://doi.org/10.1186/S12906-017-2000-0/TABLES/9

Son, N. H., Tuan, N. T., & Tran, T. M. (2022). Investigation of chemical composition and evaluation of antioxidant, antibacterial and antifungal activities of ethanol extract from *Bidens pilosa* L. *Food Science and Technology*, *42*, e22722. https://doi.org/10.1590/FST.22722

Tesfay, S. Z., Mathe, S., Modi, A. T., & Mabhaudhi, T. (2016). A Comparative Study on Antioxidant Potential of Selected African and Exotic Leafy Vegetables. *HortScience*, *51*(12), 1529–1536. https://doi.org/10.21273/HORTSCI11161-16

Tsuruta, K., Shidara, T., Miyagishi, H., Nango, H., Nakatani, Y., Suzuki, N., Amano, T., Suzuki, T., & Kosuge, Y. (2023). Anti-Inflammatory Effects of Miyako Bidens pilosa in a Mouse Model of Amyotrophic Lateral Sclerosis and Lipopolysaccharide-Stimulated BV-2 Microglia. *International Journal of Molecular Sciences 2023, Vol. 24, Page 13698*, *24*(18), 13698. https://doi.org/10.3390/IJMS241813698

U-Yatung, S., Suebsaiprom, W., Pornprom, T., & Chompoo, J. (2020). Performance of some thai weed extracts on antioxidants and atherosclerosis-related enzymes. *Agrivita*, *42*(2), 243–254. https://doi.org/10.17503/AGRIVITA.V0I0.2322

Wu, J., Wan, Z., Yi, J., Wu, Y., Peng, W., & Wu, J. (2012). Investigation of the extracts from Bidens pilosa Linn. var. radiata Sch. Bip. for antioxidant activities and cytotoxicity against human tumor cells. *Journal of Natural Medicines 2012 67:1*, *67*(1), 17–26. https://doi.org/10.1007/S11418-012-0639-X

Xin, Y.-J., Choi, S., Roh, K.-B., Cho, E., Ji, H., Weon, J. B., Park, D., Whang, W. K., & Jung, E. (2021). Anti-Inflammatory Activity and Mechanism of Isookanin, Isolated by Bioassay-Guided Fractionation from Bidens pilosa L. *Molecules*, *26*(2), 255. https://doi.org/10.3390/molecules26020255

Yan, Z., Chen, Z., Zhang, L., Wang, X., Zhang, Y., & Tian, Z. (2022). Bioactive polyacetylenes from Bidens pilosa L and their anti-inflammatory activity. *Natural Product Research*, *36*(24), 6353–6358. https://doi.org/10.1080/14786419.2022.2029432;WGROUP:STRING:PUBLICATION

Yang, H. L., Chen, S. C., Chang, N. W., Chang, J. M., Lee, M. L., Tsai, P. C., Fu, H. H., Kao, W. W., Chiang, H. C., Wang, H. H., & Hseu, Y. C. (2006). Protection from oxidative damage using Bidens pilosa extracts in normal human erythrocytes. *Food and Chemical Toxicology*, *44*(9), 1513–1521. https://doi.org/10.1016/J.FCT.2006.04.006

Yang, Y., Yu, K., & Zhang, Y. M. (2018). The Cardioprotective Effects of 4-O-(2″-O-acetyl-6″-O- P-coumaroyl-β-D-glucopyranosyl)-P-coumaric Acid (4-ACGC) on Chronic Heart Failure. *Iranian Journal of Pharmaceutical Research : IJPR*, *17*(2), 593.

Yi, J., Wu, J. G., Wu, Y. Bin, & Peng, W. (2016). Antioxidant and Anti-proliferative Activities of Flavonoids from Bidens pilosa L var radiata Sch Bip. *Tropical Journal of Pharmaceutical Research*, *15*(2), 341–348. https://doi.org/10.4314/TJPR.V15I2.17

Yuan, L. P., Chen, F. H., Ling, L., Dou, P. F., Bo, H., Zhong, M. M., & Xia, L. J. (2008). Protective effects of total flavonoids of Bidens pilosa L. (TFB) on animal liver injury and liver fibrosis. *Journal of Ethnopharmacology*, *116*(3), 539–546. https://doi.org/10.1016/J.JEP.2008.01.010

Yuniastri, R., Huzaimah, N., Estiasih, T., Martati, E., Tarmadi, D., Fatriasari, W., Arung, E. T., & Ismayati, M. (2022). *A COMPARATIVE EVALUATION OF THE ANTIOXIDANT ACTIVITY OF LOCAL PLANTS ORIGINATED FROM SUMENEP REGENCY, EAST JAVA, INDONESIA*. 87–94. https://doi.org/10.31788/RJC.2022.1558120

Abiodun, O. O., Sosanya, A. S., Nwadike, N., & Oshinloye, A. O. (2020). Beneficial effect of Bidens pilosa L. (Asteraceae) in a rat model of colitis. *Journal of Basic and Clinical Physiology and Pharmacology*, *31*(6), 1–9. https://doi.org/10.1515/jbcpp-2019-0166

Adeolu Adedapo, F. J. and A. A. (2011). *Comparison of the nutritive value and biological activities of the acetone, methanol and water extracts of the leaves of Bidens pilosa and Chenopodium album - PubMed*. Acta Poloniae Pharmaceutica ñ Drug Research, Vol. 68 No. 1 Pp. 83ñ92, 2011.

Akula, U. S., & Odhav, B. (2008). *In vitro 5-Lipoxygenase inhibition of polyphenolic antioxidants from undomesticated plants of South Africa*. *2*(9), 207–212.

Angelini, P., Matei, F., Flores, G. A., Pellegrino, R. M., Vuguziga, L., Venanzoni, R., Tirillini, B., Emiliani, C., Orlando, G., Menghini, L., & Ferrante, C. (2021). Metabolomic Profiling, Antioxidant and Antimicrobial Activity of Bidens pilosa. *Processes 2021, Vol. 9, Page 903*, *9*(6), 903. https://doi.org/10.3390/PR9060903

Arantes, D. A. C., da Silva, A. C. G., Freitas, N. M. A., Lima, E. M., de Oliveira, A. C., Marreto, R. N., Mendonça, E. F., & Valadares, M. C. (2021). Safety and efficacy of a mucoadhesive phytomedication containing curcuminoids and Bidens pilosa L. extract in the prevention and treatment of radiochemotherapy-induced oral mucositis: Triple-blind, randomized, placebo-controlled, clinical trial. *Head and Neck*, *43*(12), 3922–3934. https://doi.org/10.1002/HED.26892;PAGEGROUP:STRING:PUBLICATION

Arantes, D. A. C., da Silva, A. C. G., Lima, E. M., Alonso, E. C. P., Marreto, R. N., Mendonça, E. F., Valadares, M. C., & Batista, A. C. (2021). Biological effects of formulation containing curcuminoids and *Bidens Pilosa L*. in oral carcinoma cell line. *Brazilian Oral Research*, *35*, e063. https://doi.org/10.1590/1807-3107BOR-2021.VOL35.0063

Bastos, C. C. C., Ávila, P. H. M. de, Filho, E. X. dos S., Ávila, R. I. de, Batista, A. C., Fonseca, S. G., Lima, E. M., Marreto, R. N., Mendonça, E. F. de, & Valadares, M. C. (2016). Use of Bidens pilosa L. (Asteraceae) and Curcuma longa L. (Zingiberaceae) to treat intestinal mucositis in mice: Toxico-pharmacological evaluations. *Toxicology Reports*, *3*, 279–287. https://doi.org/10.1016/j.toxrep.2015.10.013

Chang, C. L. T., Kuo, H. K., Chang, S. L., Chiang, Y. M., Lee, T. H., Wu, W. M., Shyur, L. F., & Yang, W. C. (2005). The distinct effects of a butanol fraction of Bidens pilosa plant extract on the development of Th1-mediated diabetes and Th2-mediated airway inflammation in mice. *Journal of Biomedical Science*, *12*(1), 79–89. https://doi.org/10.1007/S11373-004-8172-X/METRICS

Chiang, Y. M., Chuang, D. Y., Wang, S. Y., Kuo, Y. H., Tsai, P. W., & Shyur, L. F. (2004). Metabolite profiling and chemopreventive bioactivity of plant extracts from Bidens pilosa. *Journal of Ethnopharmacology*, *95*(2–3), 409–419. https://doi.org/10.1016/J.JEP.2004.08.010

Chih, H. W., Lin, C. C., & Tang, K. S. (1995). Anti-inflammatory activity of Taiwan folk medicine “ham-hong-chho” in rats. *The American Journal of Chinese Medicine*, *23*(3–4), 273–278. https://doi.org/10.1142/S0192415X95000328;ISSUE:ISSUE:10.1142/AJCM.23.ISSUE-03N04;PAGEGROUP:STRING:PUBLICATION

Chipurura, B., Muchuweti, M., & Bhebhe, M. (2013). An assessment of the phenolic content, composition and antioxidant capacity of selected indigenous vegetables of Zimbabwe. *Acta Horticulturae*, *979*, 611–620. https://doi.org/10.17660/ACTAHORTIC.2013.979.66

Cortés-Rojas, D. F., Souza, C. R. F., & Oliveira, W. P. (2011). Optimisation of the extraction of phenolic compounds and antioxidant activity from aerial parts of Bidens pilosa L. using response surface methodology. *International Journal of Food Science and Technology*, *46*(11), 2420–2427. https://doi.org/10.1111/J.1365-2621.2011.02765.X

de Ávila, P. H. M., de Ávila, R. I., dos Santos Filho, E. X., Cunha Bastos, C. C., Batista, A. C., Mendonça, E. F., Serpa, R. C., Marreto, R. N., da Cruz, A. F., Lima, E. M., & Valadares, M. C. (2015). Mucoadhesive formulation of Bidens pilosa L. (Asteraceae) reduces intestinal injury from 5-fluorouracil-induced mucositis in mice. *Toxicology Reports*, *2*, 563. https://doi.org/10.1016/J.TOXREP.2015.03.003

Deba, F., Xuan, T. D., Yasuda, M., & Tawata, S. (2007). Chemical composition and antioxidant, antibacterial and antifungal activities of the essential oils from Bidens pilosa Linn. var. Radiata. *Food Control*, *19*(4), 346–352. https://doi.org/10.1016/J.FOODCONT.2007.04.011

Falowo, A. B., Muchenje, V., Hugo, A., Aiyegoro, O. A., & Fayemi, P. O. (2017). Antioxidant activities of Moringa oleifera L. and Bidens pilosa L. leaf extracts and their effects on oxidative stability of ground raw beef during refrigeration storage. *CyTA - Journal of Food*, *15*(2), 249–256. https://doi.org/10.1080/19476337.2016.1243587

Fotso, A. F., Longo, F., Djomeni, P. D. D., Kouam, S. F., Spiteller, M., Dongmo, A. B., & Savineau, J. P. (2014). Analgesic and antiinflammatory activities of the ethyl acetate fraction of Bidens pilosa (Asteraceae). *Inflammopharmacology*, *22*(2), 105–114. https://doi.org/10.1007/s10787-013-0196-2

Goudoum, A., Abdou, A. B., Ngamo, L. S. T., Ngassoum, M. B., & Mbofung, C. M. F. (2016). Antioxidant activities of essential oil of Bidens pilosa (Linn. Var. Radita) used for the preservation of food qualities in North Cameroon. *Food Science and Nutrition*, *4*(5), 671–678. https://doi.org/10.1002/FSN3.330

Horiuchi, M., & Seyama, Y. (2008). Improvement of the antiinflammatory and antiallergic activity of Bidens pilosa L. var. radiata SCHERFF treated with enzyme (Cellulosine). *Journal of Health Science*, *54*(3), 294–301. https://doi.org/10.1248/jhs.54.294

Horiuchi, M., Wachi, H., & Seyama, Y. (2010). Effects of Bidens pilosa L. var. radiata Scherff on experimental gastric lesion. *Journal of Natural Medicines*, *64*(4), 430–435. https://doi.org/10.1007/s11418-010-0426-5

Idris, O. A., Kerebba, N., Horn, S., Maboeta, M. S., & Pieters, R. (2023). Phytochemical-Based Evidence of the Health Benefits of Bidens Pilosa Extracts and Cytotoxicity. *Chemistry Africa*, *6*(4), 1767–1788. https://doi.org/10.1007/s42250-023-00626-2

Kviecinski, M. R., Felipe, K. B., Correia, J. F. G., Ferreira, E. A., Rossi, M. H., Gatti, F. de M., Filho, D. W., & Pedrosa, R. C. (2011). Brazilian Bidens pilosa linné yields fraction containing quercetin-derived flavonoid with free radical scavenger activity and hepatoprotective effects. *Libyan Journal of Medicine*, *6*(1), 1–8. https://doi.org/10.3402/LJM.V6I0.5651

Liu, J. R., Ye, Y. L., Lin, T. Y., Wang, Y. W., & Peng, C. C. (2013). Effect of floral sources on the antioxidant, antimicrobial, and anti-inflammatory activities of honeys in Taiwan. *Food Chemistry*, *139*(1–4), 938–943. https://doi.org/10.1016/j.foodchem.2013.02.015

Mashinini, P. P., Chihomvu, P., Pillay, M., & Takaidza, S. (2023). Phytochemical analysis and anti-mycobacterium activity of Bidens pilosa crude extracts. *Journal of Biotech Research*, *15*(September), 116–137.

Memon, F. Q., Yang, Y., Lv, F., Soliman, A. M., Chen, Y., Sun, J., Wang, Y., Zhang, G., Li, Z., Xu, B., Gadahi, J. A., & Si, H. (2020). *Effects of probiotics and Bidens pilosa on the performance and gut health of chicken during induced E. tenella infection*. https://doi.org/10.21203/RS.3.RS-18318/V1

Mohamed, R. S., Ramadan, M. M., Fouda, K., Ghanem, K. Z., Omara, E. A., & Abdel-Aziz, S. A. (2024). Preventive Impacts of Black Tea, Green Tea and Bidens pilosa on Renal Stone Formation in Rats: Antioxidant and Anti-inflammatory Pathways. *Egyptian Journal of Chemistry*, *67*(12), 423–432. https://doi.org/10.21608/EJCHEM.2024.317701.10337

Mota, C. M., Santiago, F. M., Cardoso, M. de R. D., Rostkowska, C., de Oliveira, T. C., Silva, D. A. de O., Pirovani, C. P., Mineo, T. W. P., & Mineo, J. R. (2019). Acetonic fraction of Bidens pilosa enriched for maturase K is able to control cerebral parasite burden in mice experimentally infected with Toxoplasma gondii. *Frontiers in Veterinary Science*, *6*(MAR), 1–13. https://doi.org/10.3389/fvets.2019.00055

Muchuweti, M., Mupure, C., Ndhlala, A., Murenje, T., & Benhura, M. A. N. (2007). Screening of antioxidant and radical scavenging activity of Vigna ungiculata, Bidens pilosa and Cleome gynandra. *American Journal of Food Technology*, *2*(3), 161–168. https://doi.org/10.3923/AJFT.2007.161.168

Nguyen, T. H. D., Vu, D. C., Hanh, P. Q. P., Vo, X. T., Nguyen, V. C., Nguyen, T. N., Nguyen, L. L. P., & Baranyai, L. (2023). Comparative analysis of phenolic content and in vitro bioactivities of Bidens pilosa L. flowers and leaves as affected by extraction solvents. *Journal of Agriculture and Food Research*, *14*, 100879. https://doi.org/10.1016/J.JAFR.2023.100879

Nxumalo, K. A., Aremu, A. O., & Fawole, O. A. (2023). Metabolite profiling, antioxidant and antibacterial properties of four medicinal plants from Eswatini and their relevance in food preservation. *South African Journal of Botany*, *162*, 719–729. https://doi.org/10.1016/J.SAJB.2023.10.008

Oduntan, A. O., Fasoyiro, S. B., Akinfasoye, J. A., Adeboyejo, F. O., & Akintoye, H. A. (2018). Antioxidant and proximate properties of underutilized vegetables in western Nigeria. *Acta Horticulturae*, *1225*, 255–260. https://doi.org/10.17660/ACTAHORTIC.2018.1225.35

Pegoraro, C. M. R., Nai, G. A., Garcia, L. A., Serra, F. de M., Alves, J. A., Chagas, P. H. N., Oliveira, D. G. de, & Zocoler, M. A. (2018). Protective effects of Bidens pilosa on hepatoxicity and nephrotoxicity induced by carbon tetrachloride in rats. *Drug and Chemical Toxicology*, *44*(1), 64–74. https://doi.org/10.1080/01480545.2018.1526182;WGROUP:STRING:PUBLICATION

Pereira, R. L. C., Ibrahim, T., Lucchetti, L., Da Silva, A. J. R., & De Moraes, V. L. G. (1999). Immunosuppressive and anti-inflammatory effects of methanolic extract and the polyacetylene isolated from Bidens pilosa L. *Immunopharmacology*, *43*(1), 31–37. https://doi.org/10.1016/S0162-3109(99)00039-9

Phiri, H., Lumai, A., Zombe, K., & Nyirenda, J. (2024). Evaluation of antioxidant activity of selected wild fruits and vegetables from Zambia. *Food and Humanity*, *3*, 100390. https://doi.org/10.1016/J.FOOHUM.2024.100390

Quaglio, A. E. V., Cruz, V. M., Almeida-Junior, L. D., Costa, C. A. R. A., & Di Stasi, L. C. (2020). Bidens pilosa (Black Jack) Standardized Extract Ameliorates Acute TNBS-induced Intestinal Inflammation in Rats. *Planta Medica*, *86*(05), 319–330. https://doi.org/10.1055/A-1089-8342

Said, W., Khattab, A. A., Hamed, S. A., Abo-Elmaaty, S. A., & Khalil, H. (2024). Identification of Bioactive and Anticancer Properties of Bidens Pilosa in-vitro Evidence. *Asian Pacific Journal of Cancer Prevention : APJCP*, *25*(10), 3551. https://doi.org/10.31557/APJCP.2024.25.10.3551

Santos Filho, E. X. dos, Arantes, D. A. C., Oton Leite, A. F., Batista, A. C., Mendonça, E. F. de, Marreto, R. N., Naves, L. N., Lima, E. M., & Valadares, M. C. (2018). Randomized clinical trial of a mucoadhesive formulation containing curcuminoids (Zingiberaceae) and Bidens pilosa Linn (Asteraceae) extract (FITOPROT) for prevention and treatment of oral mucositis - phase I study. *Chemico-Biological Interactions*, *291*, 228–236. https://doi.org/10.1016/J.CBI.2018.06.010

Shandukani, P. D., Tshidino, S. C., Masoko, P., & Moganedi, K. M. (2018). Antibacterial activity and in situ efficacy of Bidens pilosa Linn and Dichrostachys cinerea Wight et Arn extracts against common diarrhoea-causing waterborne bacteria. *BMC Complementary and Alternative Medicine*, *18*(1). https://doi.org/10.1186/S12906-018-2230-9

Singh, G., Passsari, A. K., Singh, P., Leo, V. V., Subbarayan, S., Kumar, B., Singh, B. P., lalhlenmawia, H., & Kumar, N. S. (2017). Pharmacological potential of Bidens pilosa L. and determination of bioactive compounds using UHPLC-QqQLIT-MS/MS and GC/MS. *BMC Complementary and Alternative Medicine*, *17*(1), 1–16. https://doi.org/10.1186/S12906-017-2000-0/TABLES/9

Son, N. H., Tuan, N. T., & Tran, T. M. (2022). Investigation of chemical composition and evaluation of antioxidant, antibacterial and antifungal activities of ethanol extract from *Bidens pilosa* L. *Food Science and Technology*, *42*, e22722. https://doi.org/10.1590/FST.22722

Tesfay, S. Z., Mathe, S., Modi, A. T., & Mabhaudhi, T. (2016). A Comparative Study on Antioxidant Potential of Selected African and Exotic Leafy Vegetables. *HortScience*, *51*(12), 1529–1536. https://doi.org/10.21273/HORTSCI11161-16

Tsuruta, K., Shidara, T., Miyagishi, H., Nango, H., Nakatani, Y., Suzuki, N., Amano, T., Suzuki, T., & Kosuge, Y. (2023). Anti-Inflammatory Effects of Miyako Bidens pilosa in a Mouse Model of Amyotrophic Lateral Sclerosis and Lipopolysaccharide-Stimulated BV-2 Microglia. *International Journal of Molecular Sciences 2023, Vol. 24, Page 13698*, *24*(18), 13698. https://doi.org/10.3390/IJMS241813698

U-Yatung, S., Suebsaiprom, W., Pornprom, T., & Chompoo, J. (2020). Performance of some thai weed extracts on antioxidants and atherosclerosis-related enzymes. *Agrivita*, *42*(2), 243–254. https://doi.org/10.17503/AGRIVITA.V0I0.2322

Wu, J., Wan, Z., Yi, J., Wu, Y., Peng, W., & Wu, J. (2012). Investigation of the extracts from Bidens pilosa Linn. var. radiata Sch. Bip. for antioxidant activities and cytotoxicity against human tumor cells. *Journal of Natural Medicines 2012 67:1*, *67*(1), 17–26. https://doi.org/10.1007/S11418-012-0639-X

Xin, Y.-J., Choi, S., Roh, K.-B., Cho, E., Ji, H., Weon, J. B., Park, D., Whang, W. K., & Jung, E. (2021). Anti-Inflammatory Activity and Mechanism of Isookanin, Isolated by Bioassay-Guided Fractionation from Bidens pilosa L. *Molecules*, *26*(2), 255. https://doi.org/10.3390/molecules26020255

Yan, Z., Chen, Z., Zhang, L., Wang, X., Zhang, Y., & Tian, Z. (2022). Bioactive polyacetylenes from Bidens pilosa L and their anti-inflammatory activity. *Natural Product Research*, *36*(24), 6353–6358. https://doi.org/10.1080/14786419.2022.2029432;WGROUP:STRING:PUBLICATION

Yang, H. L., Chen, S. C., Chang, N. W., Chang, J. M., Lee, M. L., Tsai, P. C., Fu, H. H., Kao, W. W., Chiang, H. C., Wang, H. H., & Hseu, Y. C. (2006). Protection from oxidative damage using Bidens pilosa extracts in normal human erythrocytes. *Food and Chemical Toxicology*, *44*(9), 1513–1521. https://doi.org/10.1016/J.FCT.2006.04.006

Yang, Y., Yu, K., & Zhang, Y. M. (2018). The Cardioprotective Effects of 4-O-(2″-O-acetyl-6″-O- P-coumaroyl-β-D-glucopyranosyl)-P-coumaric Acid (4-ACGC) on Chronic Heart Failure. *Iranian Journal of Pharmaceutical Research : IJPR*, *17*(2), 593.

Yi, J., Wu, J. G., Wu, Y. Bin, & Peng, W. (2016). Antioxidant and Anti-proliferative Activities of Flavonoids from Bidens pilosa L var radiata Sch Bip. *Tropical Journal of Pharmaceutical Research*, *15*(2), 341–348. https://doi.org/10.4314/TJPR.V15I2.17

Yuan, L. P., Chen, F. H., Ling, L., Dou, P. F., Bo, H., Zhong, M. M., & Xia, L. J. (2008). Protective effects of total flavonoids of Bidens pilosa L. (TFB) on animal liver injury and liver fibrosis. *Journal of Ethnopharmacology*, *116*(3), 539–546. https://doi.org/10.1016/J.JEP.2008.01.010

Yuniastri, R., Huzaimah, N., Estiasih, T., Martati, E., Tarmadi, D., Fatriasari, W., Arung, E. T., & Ismayati, M. (2022). *A COMPARATIVE EVALUATION OF THE ANTIOXIDANT ACTIVITY OF LOCAL PLANTS ORIGINATED FROM SUMENEP REGENCY, EAST JAVA, INDONESIA*. 87–94. https://doi.org/10.31788/RJC.2022.1558120
